# Analysis of Tyrosine Kinase Inhibitor-Mediated Decline in Contractile Force in Rat Engineered Heart Tissue

**DOI:** 10.1371/journal.pone.0145937

**Published:** 2016-02-03

**Authors:** Fabian Jacob, Amina Y. Yonis, Friederike Cuello, Pradeep Luther, Thomas Schulze, Alexandra Eder, Thomas Streichert, Ingra Mannhardt, Marc N. Hirt, Sebastian Schaaf, Justus Stenzig, Thomas Force, Thomas Eschenhagen, Arne Hansen

**Affiliations:** 1 Department of Experimental Pharmacology and Toxicology, Cardiovascular Research Center, University Medical Center Hamburg-Eppendorf, Hamburg, Germany, DZHK (German Centre for Cardiovascular Research), partner site Hamburg/Kiel/Lübeck, Hamburg, Germany; 2 Molecular Medicine Section, National Heart and Lung Institute, Faculty of Medicine, Imperial College London, London, United Kingdom; 3 Department of Clinical Chemistry/Central Laboratories, University Medical Center Hamburg-Eppendorf, Hamburg, Germany; 4 Center for Translational Medicine, Cardiology Division, Temple University School of Medicine, Philadelphia, Pennsylvania, 19140, United States of America; Seoul National University, REPUBLIC OF KOREA

## Abstract

**Introduction:**

Left ventricular dysfunction is a frequent and potentially severe side effect of many tyrosine kinase inhibitors (TKI). The mode of toxicity is not identified, but may include impairment of mitochondrial or sarcomeric function, autophagy or angiogenesis, either as an on-target or off-target mechanism.

**Methods and Results:**

We studied concentration-response curves and time courses for nine TKIs in three-dimensional, force generating engineered heart tissue (EHT) from neonatal rat heart cells. We detected a concentration- and time-dependent decline in contractile force for gefitinib, lapatinib, sunitinib, imatinib, sorafenib, vandetanib and lestaurtinib and no decline in contractile force for erlotinib and dasatinib after 96 hours of incubation. The decline in contractile force was associated with an impairment of autophagy (LC3 Western blot) and appearance of autophagolysosomes (transmission electron microscopy).

**Conclusion:**

This study demonstrates the feasibility to study TKI-mediated force effects in EHTs and identifies an association between a decline in contractility and inhibition of autophagic flux.

## Introduction

The side effect profile of TKIs differs substantially from conventional anti-cancer drugs. However, a number of side effects were revealed and structural (in contrast to proarrhythmic) cardiotoxicity is frequent among these [1,2]. The relatively frequent occurrence of TKI-associated cardiotoxicity was not anticipated since highly proliferative tumor cells and terminally differentiated cardiomyocytes display fundamental differences in cellular biology. More detailed studies, however, revealed that the underlying molecular mechanisms of cancer cell- and cardiomyocyte-biology display a substantial degree of similarity [3]. In particular, high-energy consumption and active cellular recycling pathways (autophagy) are peculiar characteristics of both.

The first indication for structural cardiotoxicity mediated by TKIs appeared when patients treated with imatinib developed heart failure [4]. Histologically, mitochondrial abnormalities and cytoplasmic vacuoles were detected and linked kinase inhibition with mitochondrial dysfunction. Follow up studies in cultured cardiomyocytes and animal models provided further evidence that imatinib was associated with mitochondrial insufficiency resulting in cytochrome c-release and compromised energy generation, decline in ATP concentrations and cell death. Retroviral gene transfer of an imatinib-resistant mutant of c-ABL partially rescued this toxicity, suggesting that c-ABL is involved in the mechanism of toxicity [4,5]. Morphologically imatinib cardiotoxicity in rats was characterized by cytoplasmic vacuolization and myofibrillar loss [6]. Further studies in rodents and zebrafish identified inhibition of AMPK and RAF 1/BRAF as key cardiotoxic mechanisms for sunitinib and sorafenib, respectively [7,8].

Modulation of autophagy has been proposed as one potential mechanism of kinase inhibitor mediated action/side effects [9]. Autophagy is a catabolic process that leads to the sequestration and degradation of misfolded proteins and cellular organelles. The initiation of autophagy results in the generation of phagophores. This process involves cleavage of microtubule-associated protein 1 light chain 3 (LC3) by autophagin-4 (Atg-4) to generate LC3-I. Through the action of Atg-3 and Atg-7, LC3-I is further processed to LC3-II. Finally, autophagosomes are formed and fuse with lysosomes, resulting in degradation of cargo material.

In part, the poor understanding of structural cardiotoxicity mediated by kinase inhibitors is a consequence of the lack of good animal and *in vitro* models and the relatively low frequency of this side effect in patients, suggesting that often risk factors must come together to cause this problem. In fact, the cardiotoxicity of kinase inhibitors has not been discovered during preclinical drug development, but only in clinical trials with these compounds. Studies in rodents suggest that it is challenging to demonstrate left ventricular dysfunction upon treatment with sunitinib in the absence of pressure overload [7,10]. This could indicate that compensatory mechanisms of the organism contribute to the low sensitivity of this model. Zebrafish models may have a higher sensitivity and have proven to be useful to demonstrate ventricular dysfunction of sunitinib and sorafenib [8] but species differences may limit wide-spread use.

The engineered heart tissue (EHT) model is a three-dimensional, force-generating cardiac tissue model, generated with high levels of standardisation and reproducibility from dissociated heart cells and fibrin matrix between flexible silicone posts [11]. In this study, we analyzed the effect of nine small molecule kinase inhibitors on EHTs from neonatal rat cardiomyocytes (NRCM) by analyzing contractile function, immunohistology, transmission electron microscopy, and clinical chemistry.

## Materials and Methods

### Generation of EHTs

EHT were generated as previously described [11]. Briefly, ventricular heart cells from neonatal Wistar and Lewis rats (balanced numbers, postnatal day 0 to 3) were isolated by fractionated DNase/Trypsin digestion. Direct comparisons between Wistar- and Lewis-EHTs did not reveal systematic differences (unpublished own data). Heart cells were resuspended in a mastermix containing Dulbecco's Modified Eagle's Medium (DMEM) and fibrinogen (5 mg/ml). Agarose casting molds were created with custom-made teflon spacers and liquid agarose (2% in phosphate buffered saline, PBS). After solidification teflon spacers were removed and silicone racks were placed on 24 well plates, ensuring that pairs of silicone posts reached into the casting molds. 97 μl of the mastermix (containing 0.41x10^6^ cells) were briefly mixed with 3 μl of thrombin (3 U/ml) and pipetted into the casting molds. After two hours fibrin was polymerized and formed a gel between the silicone posts. Silicone racks were transferred to new 24 well plates and were maintained under cell culture conditions for two weeks (37°C, 7% CO_2_ and 40% O_2_). EHT medium consisted of DMEM (Biochrom F0415), 10% horse serum (Gibco 26050), 2% chick embryo extract, 1% penicillin/streptomycin (Gibco 15140), insulin (10 μg/mL, Sigma-Aldrich I9278) and aprotinin (33 μg/mL, Sigma-Aldrich A1153) and was changed on Mondays, Wednesdays and Fridays. Development of contractile force was monitored by video-optical recording and analysis as recently described [11–13]. Fibroblast EHTs were generated by plating dissociated neonatal rat heart cells on 0.1% gelatine–coated cell culture flasks. They were expanded for four passages by 1:3 trypsin-based split of confluent cell layers (medium: DMEM Biochrom F0415), 10% fetal calf serum, 1% penicillin/streptomycin, 1% L-glutamine). Dissociated passage 4-cells were used to generate fibroblast EHTs according to the heart cell EHT protocoll using 0.41x10^6^ cells per construct. TKI incubation was started 3 days after casting. All experimental procedures were reviewed and approved by Ethics Committee, University Hamburg (approval number ORG238).

### Analysis of TKIs

TKIs were purchased from LC Laboratories (dasatinib D-3307, erlotinib E-4007, gefitinib G-4408, imatinib I-5508, lapatinib L-4899, lestaurtinib L-6307, sorafenib S-8599, sunitinib S-8803, vandetanib V-9402) and solubilized in dimethyl sulfoxide (DMSO). Four logarithmically-diluted concentrations per TKI were analyzed (each group n = 4). DMSO was diluted with the TKI, and the DMSO concentration in vehicle control was the same as in the group with the highest TKI/DMSO concentration. TKI DMSO stock concentrations were adjusted according to maximal solubility, resulting in maximal study concentrations of 10-100x total therapeutic plasma concentration (TPC). Exceptions were dasatinib and lapatinib with maximal study concentrations of 250x and 136xTPC, respectively. Lestaurtinib was studied at a maximal concentration of 100 μM (12xTPC) despite higher DMSO solubility because pilot experiments indicated a high toxicity (e.g. decline in contractile force) for lestaurtininb in this model. Effects of TKIs were always compared with the respective vehicle control (1% DMSO for erlotinib, lapatinib, vandetanib, lestaurtinib and 0.1% DMSO for dasatinib, gefitinib, sunitinib, imatinib and sorafenib). S1 Table lists the TKI concentrations used in this study and the corresponding TPC. TKI concentrations were adjusted in EHT medium and the effect on EHT contractility (systolic force development) was analyzed. Video-optical recordings in the presence of TKI and controls were performed after 2, 48 and 96 hours of incubation. Cell culture medium was sampled after 48 and 96 hours for analysis of lactate dehydrogenase (LDH). After 96 hours, EHT were further processed for histological, transmission electron microscopic and western blot analysis. S1 Fig illustrates the experimental design of this study and the relationship between study concentration and TPC. For the time series experiments, EHTs were sampled daily between 24 and 96 hours of incubation and processed in analogy to endpoint analysis after 96 hours.

### Clinical chemistry

Analysis of LDH- and CK-activity in cell culture medium supernatant was performed in EHT medium containing phenol red-free DMEM (Gibco, 11880–028). Medium was sampled after 48 and 96 hours and centrifuged (5 min at 300 g, Eppendorf 5415R). Supernatant was transferred into a new tube and stored at -20°C. Samples were thawed, centrifuged (10 s, 13200 g, Eppendorf 5415R) and analyzed (Roche P-Module).

### Histology

EHTs were washed with pre-warmed PBS twice for 5 minutes and fixed in 4% formalin overnight. After a brief wash with tris-buffered saline (TBS), EHTs were detached from silicone posts and embedded in 4% agarose to adjust the position in the paraffin block. Dehydration was performed with a Leica ASP 300s instrument. After paraffin embedding, 4 μm longitudinal sections were prepared. Sections for staining and analysis had a distance of approximately 100 μm to the tangential cut of the tissue. This procedure accounts for the inhomogeneity of cell density throughout EHTs and allowed direct comparisons. Anti-α-sarcomeric actin, DakoCytomation M0874, 1:200 and active caspase-3, R&D Systems AF835, 1:100, staining was performed with a Benchmark XT instrument (Ventana Medical Systems).

### Transmission electron microscopy

EHTs were incubated for 10 min in 2,3-butanedione monoxime (30 mM, PBS) to relax the myofibrils. The EHTs were fixed in glutaraldehyde (Agar scientific AGR1010, 2.5% in PBS, 1 mM CaCl_2,_ pH 7.4) overnight at 4°C. Samples were further fixed and dehydrated by the EMP-5160 Automatic Tissue Processor (RMC products). EHTs were embedded in glycid ether (Serva). Semithin and ultrathin slices with longitudinal orientation were prepared with an ultramicrotome (Ultracut E, Reichert-Jung). The ultrathin slices were transferred to copper/rhodium grids (Cu/Rh, 3 mm, 200 mesh) and stained with uranyl acetate and lead citrate. The grids were observed with a transmission electron microscope (Jeol 1200 EX) fitted with a Tietz FastScan CCD camera (1024x1024 pixels). For stereological analysis of the organelles in the cells, mosaics were assembled from overlapping images recorded at 3000x magnification. Mosaics were composed of 4–9 single overlapping EM pictures of a representative area (~1000–1500 μm^2^) in the EHT. For the stereological analysis, cross-sectional area for myofibrils, healthy mitochondria, cytoplasm, disarray/autophagy was marked and quantified (Image J).

### Western blot

To measure autophagic flux, EHTs were cultured in the absence or presence of the lysosome-inhibitor bafilomycin A (Sigma-Aldrich B1793, 100 nM, 2 hours). EHT were removed from silicone posts, snap-frozen in liquid nitrogen and stored at -80°C. Protein extraction was performed with 60 μl extraction buffer per EHT (M-PER, Thermo Scientific #78501, Mini-Complete protease inhibitor, Roche 11837580001; PhosSTOP, Roche 04906837001). Tissue was lysed with the Qiagen Tissue Lyser. Proteins were separated by standard sodium dodecyl sulfate-polyacrylamide gel electrophoresis (SDS-PAGE) and blotted onto polyvinylidene fluoride (PVDF) membrane in a semidry blotting chamber (Hoefer Semiphor, Semidry transfer unit). The membranes were blocked in milk powder (5%, Tris-Buffered Saline plus Tween 20, 0.1% [TBST]). The membranes were washed (3 times, 5 min, TBST) and incubated on the roller mixer (Stuart) with the primary antibody (LC3, Novus Biologicals NB100-2331, 1:500) at 4°C overnight. The membranes were washed (3 times, 10 min, 0.1% TBST), incubated with the secondary antibody for one hour at room temperature (RT), washed again (3 times for 5 min in TBST 0.1%) incubated with *chemiluminescence* substrate (Pierce ECL Western blotting substrate, Thermo scientific 32106) and exposed to X-ray film. Bands were quantified with a Syngene Chemidoc.

### Statistical analysis

Results are presented as means±SEM. All statistical tests were performed in GraphPad Prism version 5.02. In detail, one/two-way ANOVA and Bonferroni's or Dunnett's post-test was used. P-values <0.05 were considered statistically significant and indicated in the graphs (*).

## Results

**Fig 1:**
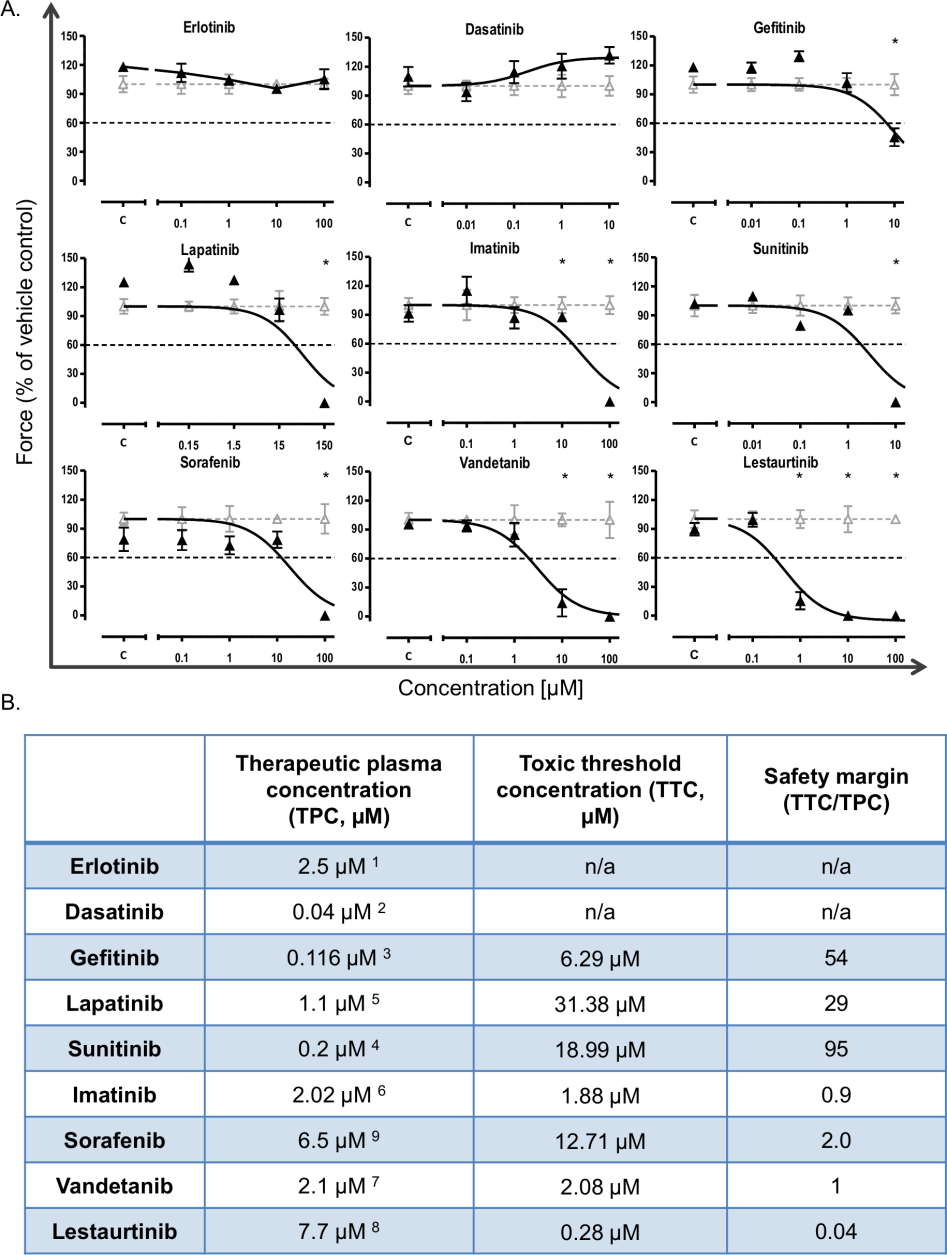
TKI effect on EHT contractility. (A) Depiction of concentration-effect curves (curve-fitted) of 9 TKIs after 96 hours of TKI incubation (▲), normalized to vehicle control (Δ). The toxic threshold (black dashed line) is defined as a decline in contractile force of >40% vs. baseline (BL). Mean values ± SEM; n = 4; *p<0.05 vs. baseline, two-way ANOVA and Bonferroni's multiple comparison post-test. (B) Total therapeutic plasma concentration (TPC), toxic threshold concentration (TTC: TKI concentration leading to ≥40% reduction in EHT contractile force) and safety margin (SM: TTC/TPC), n/a: not applicable.

EHTs started to beat coherently 7–10 days after casting. Experiments were started at day 14 when contractile force had reached a stable level, average force value was 0.17 mN ± 0.03 across all analysis at this point (mean ± SD). After addition of TKI at 4 logarithmic concentrations or DMSO (0.1 or 1%), contractile force was monitored in the presence of TKIs/DMSO for 96 hours (Fig 1A). Pilot experiments indicated that the onset of decline in contractility was rapid for some TKIs. Accordingly, three recording time points were defined: 2, 48 and 96 hours of incubation. Over this period, control and DMSO-treated EHTs showed stable contractile force (Fig 1A, S2 Fig). Erlotinib and dasatinib were not associated with reduction in contractile force at any concentration studied. In contrast, gefitinib (10 μM, 86xTPC), lapatinib (150 μM, 136xTPC), sunitinib (10 μM, 50xTPC), imatinib (100 μM, 50xTPC), and sorafenib (100 μM, 15xTPC), vandetanib (10, 100 μM, 5xTPC, 50xTPC) and lestaurtinib (1, 10, 100 μM, 0.12xTPC, 1.2xTPC, 12xTPC) led to a significant decline in contractile force. The decline occurred rapidly (after 2 hours of incubation) for vandetanib (100 μM, 50xTPC), lestaurtinib (100 μM, 12xTPC) and sorafenib (100 μM, 15xTPC) and delayed for all other conditions (Fig 1A, S2 Fig). Based on the variability of contractile force development under control conditions across all time points a threshold of >40% reduction in contractile force was defined as a toxic effect. Accordingly, toxic threshold concentrations (TTC) and safety margins (SM: TTC/therapeutic plasma concentration, TPC) were defined for all TKIs and listed in Fig 1B. We also analyzed frequency, contraction and relaxation time but did not identify additional concentration- and time-dependent effects which were not related to changes in contractile force (S2 Fig) except for a prolongation of relaxation time with erlotinib. This could indicate that erlotinib is inhibiting repolarising potassium channels since prolongation of repolarisation leads to prolongation of relaxation in the rat EHT system [14].

Lactate dehydrogenase (LDH) and creatin kinase (CK) were measured in cell culture medium after 48 and 96 hours of incubation at the highest two to three TKI concentrations (S3 Fig). A minimal but statistically significant increase in LDH activity was determined after 48 h for 0.1% DMSO and after 48 and 96 hours for 1% DMSO. Compared to vehicle control, no increase in LDH activity was detected for dasatinib, erlotinib, gefitinib and sunitinib. After 48 hours, a significant and substantial increase in LDH was determined for vandetanib (100 μM, 50xTPC, 4.8 fold), lestaurtinib (100 μM, 12xTPC, 4.6 fold) and lapatinib (150 μM, 136xTPC, 4.1 fold). LDH was modestly increased by sorafenib (100 μM, 15xTPC, 1.8 fold) and lestaurtinib (10 μM, 1.2xTPC, 1.5 fold) and slightly elevated by imatinib (100 μM, 50xTPC, 1.3 fold). LDH elevations were accentuated after 48 hours and declined after 96 hours of TKI incubation except for vandetanib, which increased from 48 to 96 hours. CK activity was significantly higher in vehicle controls (0.1% DMSO: 48 h, 1.4 fold; 1% DMSO: 48 h, 3.6 fold, 96h, 1.9 fold) than in controls (S3 Fig). Compared to vehicle control, lapatinib (150 mM, 136xTPC, 1.1 fold), sunitinib (10 μM, 50xTPC, 1.4 fold), lestaurtinib (100 μM, 12xTPC, 1.2 fold), sorafenib (100 μM, 15xTPC, 2.1 fold) and vandetanib (100 μM, 50xTPC, 1.5 fold) resulted in increased CK activity after 48 hours. After 96 hours lower concentrations of sunitinib (1 μM, 2 fold), lestaurtinib (10μM, 2 fold, 1 μM, 1.5 fold), and vandetanib (10 μM, 2 fold) as well as gefitinib (10 μM, 86xTPC, 1.6 fold) and erlotinib (100 μM, 40xTPC, 1.7 fold, 10 μM, 1.6 fold) led to increased CK activity (S3 Fig).

**Fig 2:**
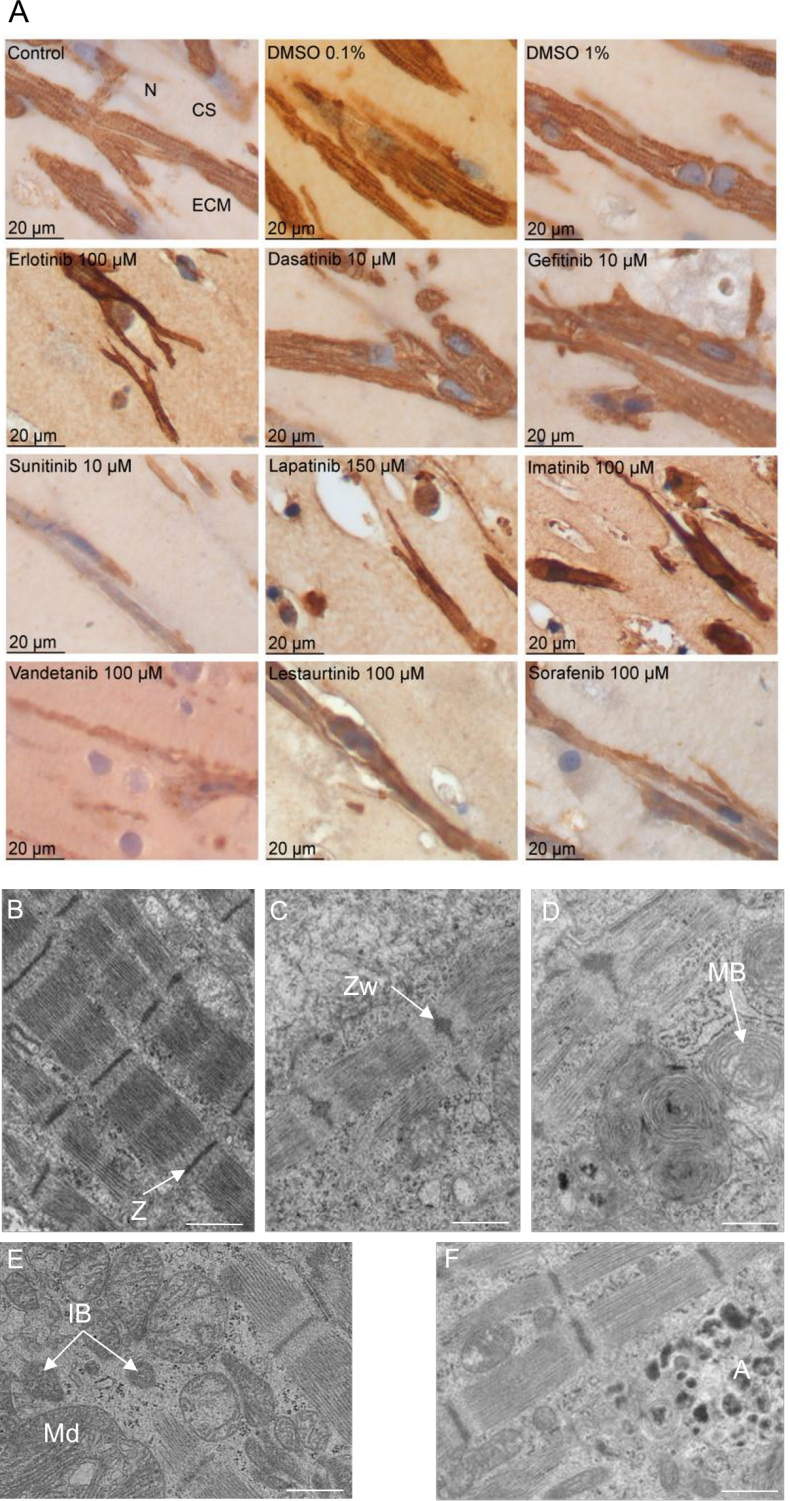
(A) α-sarcomeric actin staining of longitudinal EHT sections in the presence of TKIs and controls after 96 hours of incubation. Cross-striation (CS), extracellular matrix (ECM), nucleus (N). (B-F) Representative images of EM alterations in EHTs in the presence of TKIs. (B) Regularly structured sarcomere with Z-band (Z, 0.1% DMSO); (C) sarcomere with sarcomeric disarray, widened Z-band, (Zw, imatinib 10 μM); (D) mutilamellar bodies (MB, imatinib 10 μM); (E) mitochondria with structural defects (Md), vacuoles and inclusion bodies (IB, sunitinib 1 μM); (F) autophagy (A, vandetanib 10 μM), scale bar 1 μm.

Longitudinal sections of EHTs incubated with the highest concentration of TKIs for 96 hours were stained with antibodies against α-sarcomeric actin (Fig 2A). Sections from controls and DMSO-treated EHTs showed a dense network of aligned, α-sarcomeric actin-positive cells without apparent differences between the three groups. Dasatinib-treated EHTs did not differ from controls and erlotinib (100 μM, 40xTPC)- and gefitinib (10 μM, 86xTPC)-treated EHTs showed moderate alterations (lower cell density in erlotinib, reduction of cross-striation). In contrast, in the presence of imatinib (100 μM, 50xTPC), lapatinib (150 μM, 136xTPC), sunitinib (10 μM, 50xTPC), lestaurtinib (100 μM, 12xTPC), sorafenib (100 μM, 15xTPC) or vandetanib (100 μM, 50xTPC), the cellular density and α-sarcomeric actin-positivity cells was substantially reduced.

Due to the high-grade immunohistological alterations in the presence of the highest TKI concentration, electron microscopic longitudinal sections of the second highest TKI concentrations were analyzed for gefitinib (1 μM, 8.6xTPC), imatinib (10 μM, 5xTPC), lapatinib (15 μM, 13.6xTPC), sunitinib (1 μM, 5xTPC), lestaurtinib (10 μM, 1.2xTPC), sorafenib (10 μM, 1.5xTPC) and vandetanib (10 μM, 5xTPC) and the highest concentration for erlotinib (100 μM, 40xTPC) and dasatinib (10 μM, 250xTPC). Important findings were sarcomeric -, Z-line—and mitochondrial alterations and increased autophagy. Representative images are depicted in Fig 2B–2F. Sarcomeric alterations consisted of a reduction in the number of parallel sarcomeric bundles, loss of symmetry, reduced alignment, condensation and loss of regularity of Z-lines. Markers for mitochondrial abnormalities were structural irregularities, inclusion of vacuoles and foreign bodies and the frequency of transformation to multilamellar bodies. Autophagy was assessed by the appearance of autophagolysosomes and residual bodies. The most peculiar difference between controls and TKI-EHTs was detected for markers of autophagy (autophagolysosomes and residual bodies), suggesting that this is a morphological marker of TKI effect.

**Fig 3:**
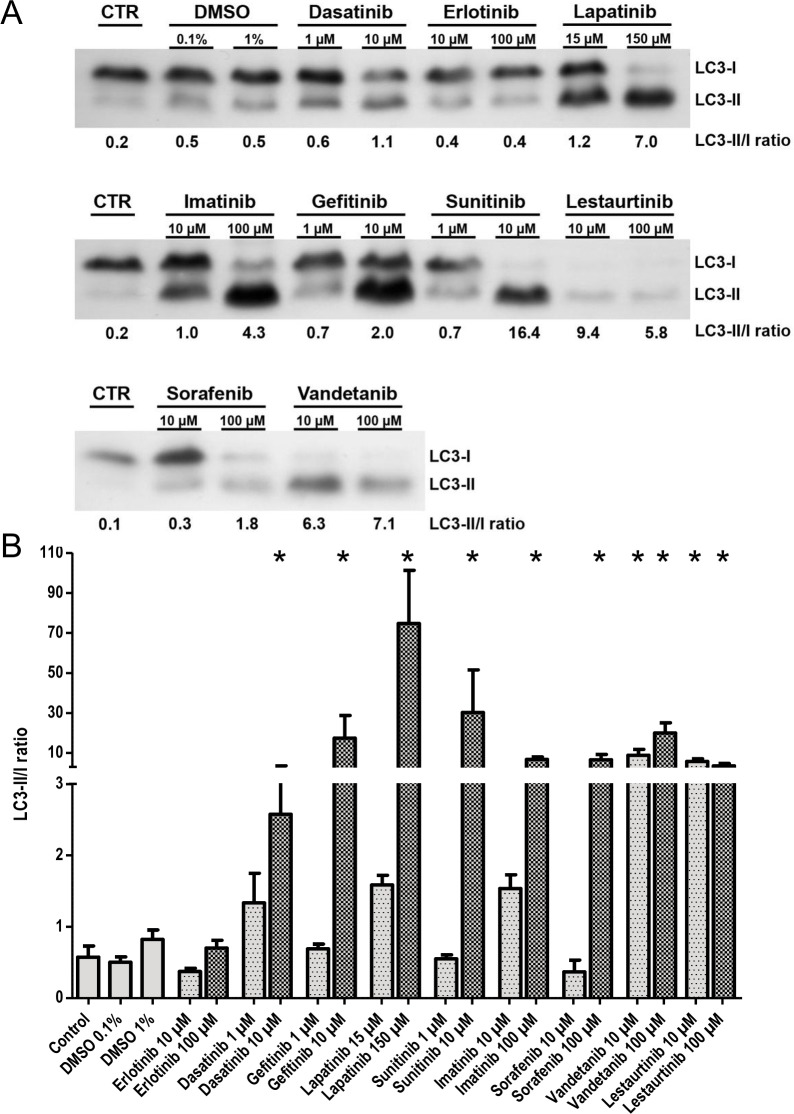
(A) LC3-II/I ratio Western blot of EHTs after 96 hours incubation: (vehicle) control or TKIs at two concentrations as indicated. LC3-I and -II band densities were quantified and LC3-II/I ratios were calculated. (B) Quantitative analysis of LC3-II/I ratios. Results are presented as means±SEM, n = 13 (controls), n = 3–5 (TKIs). One-way ANOVA and Dunnett's post-test (compared to vehicle control) were used. P-values <0.05 were considered statistically significant and indicated in the graphs (*).

Autophagy was further analyzed by immunoblot analysis for LC3-I, -II in EHTs after 96 hours of incubation with vehicle control (DMSO) or TKIs. Representative Western blots and statistical analysis are shown in Fig 3. Time matched controls and vehicle controls (0.1%, 1% DMSO) displayed a strong LC3-I and weak LC3-II band. LC3-II/I ratios were 0.6, 0.5 and 0.8, respectively. TKIs led to a concentration-dependent increase in LC3-II/I ratio. This increase was low and not significant for erlotinib (10/100 μM; 0.4, 0.7). Moderate-strong effects were observed with dasatinib (1/10 μM; 1.3, 2.6), gefitinib (1/10 μM; 0.7, 17.4), lapatinib (15/150 μM; 1.6, 74.7), sunitinib (1/10 μM; 0.6, 30.2), imatinib (10/100 μM; 1.5, 6.8) and sorafenib (10/100 μM; 0.6, 6.6) resulting in significantly higher LC3-II/I ratios for the higher concentration for each TKI. The strongest effects were demonstrated for vandetanib (10/100 μM; 8.9, 20.0) and lestaurtinib (10/100 μM; 5.8, 3.7) with significantly increased LC3-II/I ratios for both concentrations. All TKI effects on contractility, LDH- and CK-release and LC3-II/I ratio are summarized in Table 1.

**Table 1:** Correlation of biochemical (LDH, CK) or autophagic (LC3-II/I) markers with contractility of EHTs. Significant increase or decline at any time point is marked by +. Correlation of LC3-II/I ratio with contractility is higher (Phi coefficient φ = +0.894) than LDH with contractility (φ = +0.798) or CK with contractility (φ = +0.707).

Compound	Concentration	LDH increase	CK increase	LC-3 II/I increase	Contractility decline
Erlotinib	10 μM	-	+	-	-
	100 μM	-	+	-	-
Dasatinib	1 μM	-	-	-	-
	10 μM	-	-	+	-
Gefitinib	1 μM	-	-	-	-
	10 μM	-	+	+	+
Lapatinib	15 μM	-	-	-	-
	150 μM	+	+	+	+
Sunitinib	1 μM	-	+	-	-
	10 μM	-	+	+	+
Imatinib	10 μM	-	-	-	-
	100 μM	+	+	+	+
Sorafenib	10 μM	-	-	-	-
	100 μM	+	+	+	+
Vandetanib	10 μM	+	+	+	+
	100 μM	+	+	+	+
Lestaurtinib	10 μM	+	+	+	+
	100 μM	+	+	+	+

**Fig 4:**
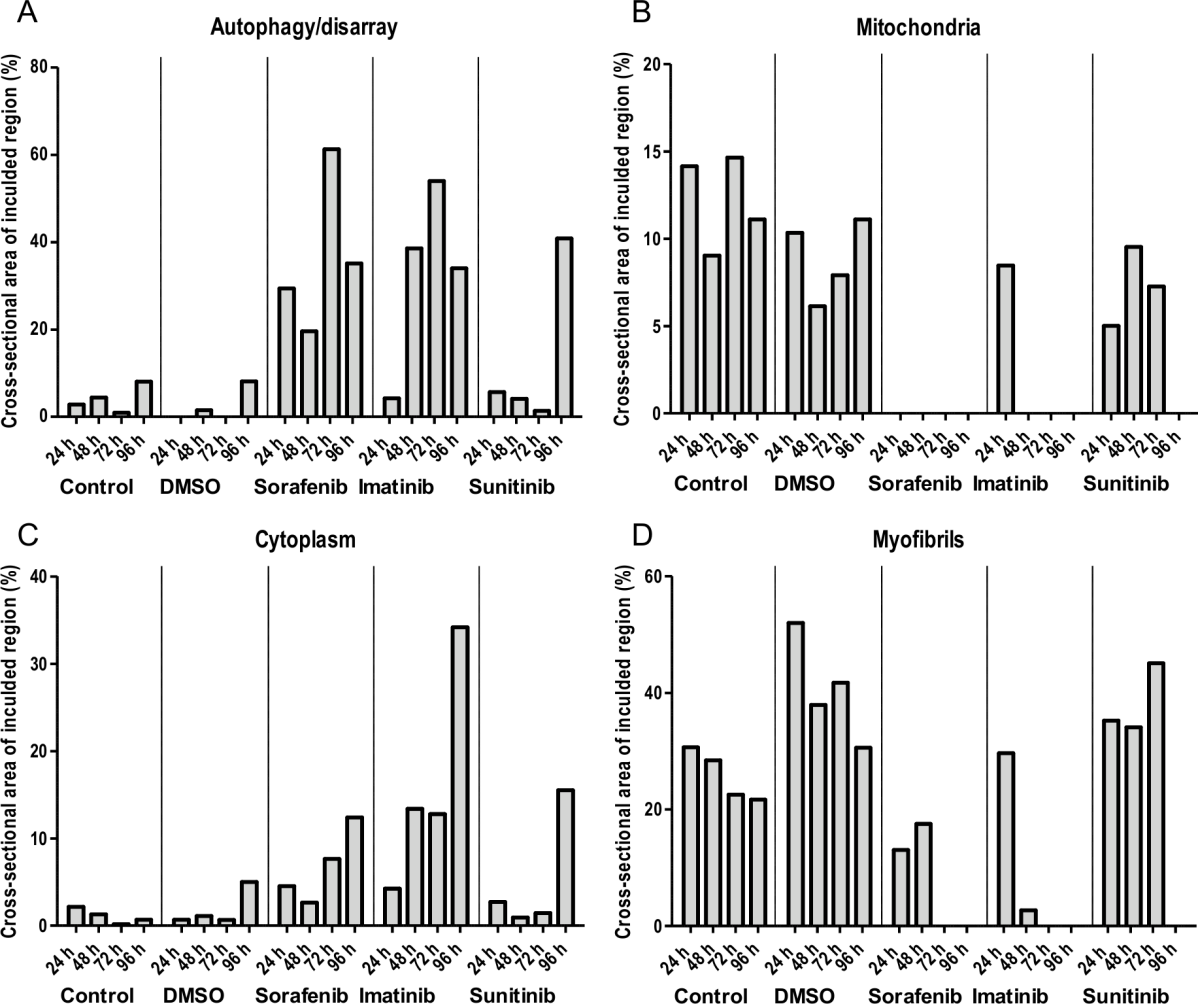
Quantification of cross-sectional area of included region (%) for disarray/autophagy (A), mitochondria (B), cytoplasma (C) and myofibrils (D) in mosaic transmission electron microscopy images after 24, 48, 72 and 96 hours of incubation under (vehicle) control conditions and with sorafenib (100 μM), imatinib (100 μM) and sunitinib (10 μM). Mosaic TEM images are shown in S4–S7 Figs. EM alterations are described in S2–S6 Tables.

**Fig 5:**
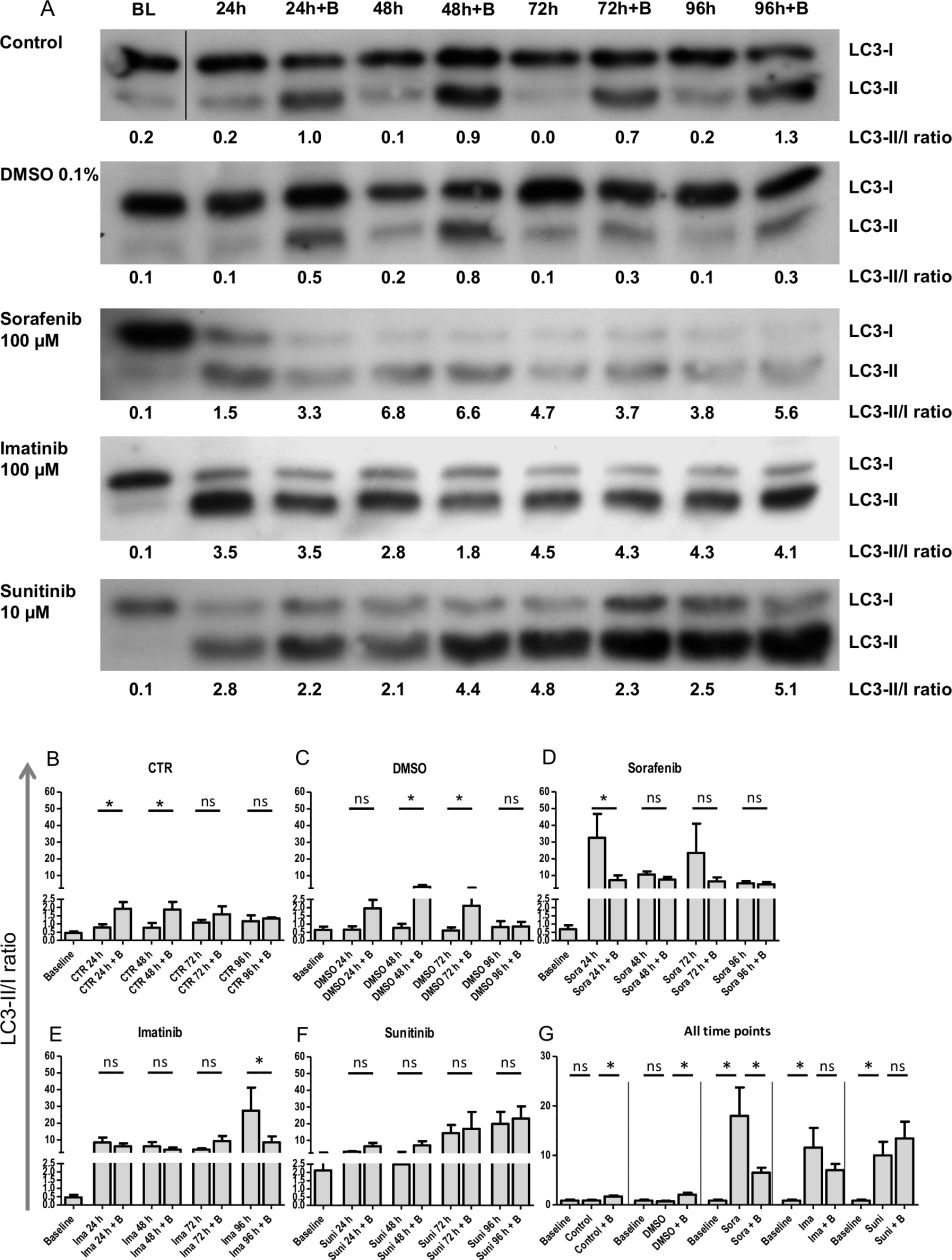
LC3 Western blot analysis of heart cell EHT and cardiac fibroblast EHT after 24–96 hours of TKI incubation in the absence and presence of bafilomycin A (B) (100 nM, 2 hours incubation). LC3 band densities were quantified and LC3–II/I ratios were calculated. (A) Representative Western blot for heart cell EHT analysis, (B-G) Quantitative analysis of LC3-II/I ratio in heart cell EHTs (B-G) and fibroblast EHT (H-M). (G, M) Comparison of LC3-II/I ratio of all time points for heart cell EHTs (G) and cardiac fibroblast EHTs (M). Results are presented as means±SEM. (B-F), (H-L): n = 3–4; (G, M): n = 16–20.One-way ANOVA and Bonferroni's post-test was used to analyze statistical significance. P-values <0.05 were considered statistically significant and indicated in the graphs (*), ns: not significant.

**Fig 6:**
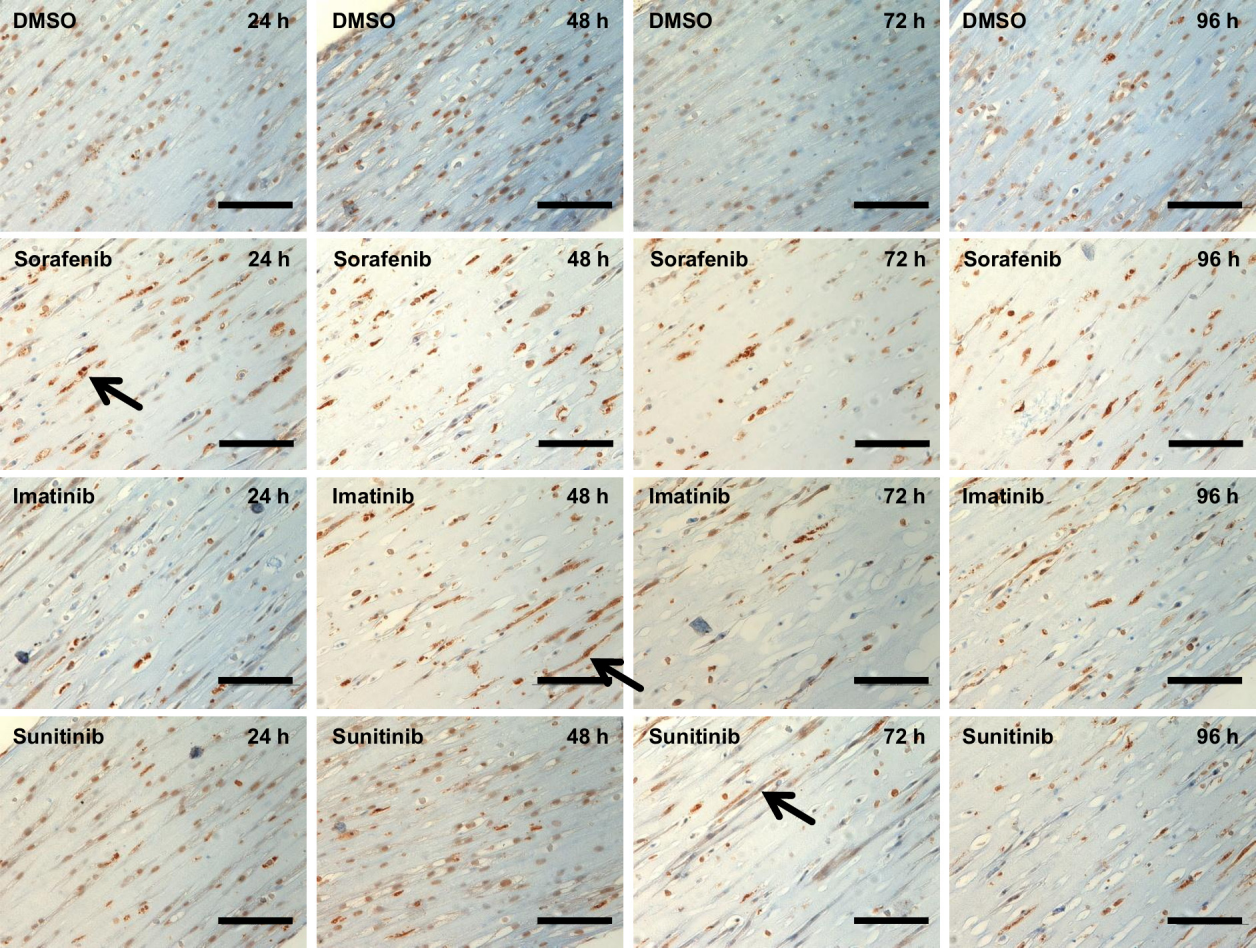
Active caspase-3 immunohistochemistry of time series (24 h-96 h) for vehicle control DMSO 0.1% (DMSO), sorafenib (100 μM), imatinib (100 μM), and sunitinib (10 μM) on heart cell EHTs. Arrows indicate cytoplasmatic caspase-3 staining. Please note nuclear caspase staining for vehicle control interpreted as fixation artifact. Scale bar 100 μm.

To dissect the kinetics of microstructural alterations, a time series for 96 hours was performed with sorafenib (100 μM), imatinib (100 μM) and sunitinib (10 μM). These three TKIs were chosen because all lead to reduction of contractile force but with different kinetics (sorafenib: 2 hours, sunitinib: 48 hours, imatinib: 96 hours) and additional aspects of their modes of cardiotoxicity were described recently. Samples for transmission electron microscopy and Western immunoblot analysis (+/- bafilomycin A) were harvested at each sampling time point. One EHT per condition and time point was subjected to transmission electron microscopy analysis. The results are summarized in S2–S6 Tables. Mosaics composed of 4–9 single EM pictures are illustrated in S4–S7 Figs. They describe well-defined sarcomeres and mitochondria with little disarray and intact structures for (vehicle) control conditions. Activity of autophagy, determined by the number of autophagolysosomes and residual bodies was low. The changes did not show incremental tendencies over time. In contrast, sorafenib, imatinib and sunitinib led to a substantial increase in sarcomeric disarray, damaged mitochondria and autophagy activity. The onset of these alterations was 24–48 hours for sorafenib and imatinib and 96 hours for sunitinib. Quantitative analyses of these alterations are expressed as percentage of cross-sectional area and shown in Fig 4. These results were complemented by immunoblot analysis for LC3-II/I of heart cell and cardiac fibroblast EHTs. Representative Western blot analysis and statistical analysis are shown in Fig 5. For heart cell EHTs LC3-II/I ratio values were unchanged for control and vehicle control conditions with maximal LC3-II/I ratio values of 0.9 and 0.7, respectively (Fig 5G). LC3-II band density increased in the presence of bafilomycin A resulting in LC3-II/I ratio values of 1.6 and 2.0. In contrast, imatinib, sorafenib and sunitinib led to a shift and enhancement of the LC3 II band and an increase of the LC3-II/I ratio (18.0, 11.5, 10.0; Fig 5G) with onset already after 24 hours (24 hours– 96 hours: sorafenib: 32.5, 10.6, 23.1, 5.3; imatinib: 8.5, 6.1, 4.2, 27.4; sunitinib: 3.2, 2.5, 14.4, 19.9) without additional bafilomycin A effect (Fig 5A–5G). To discriminate if this effect is specific to cardiomyocytes, EHTs were generated from the fibroblast fraction and analyzed accordingly. This analysis revealed that fibroblast EHTs show a very similar pattern ([vehicle] control: low LC3-II/I ratio (1.3, 1.2), bafilomycin A sensitivity (6.5, 7.0), sorafenib, imatinib, sunitinib: high LC3-II/I ratio (6.8, 13.6, 16.2), bafilomycin A insensitivity (8.9, 10.9, 10.3; Fig 5H–5M, values shown in Fig 5M). Active caspase-3 immunohistochemistry was performed on the same series, demonstrating no cyotoplasmic caspase-3 positivity or cellular rarefication in the 96 hour time course for vehicle control. In contrast, cytoplasmic caspase-3 positivity and cellular rarefication was present in TKI samples (Fig 6). In addition, nuclear caspase-3 positivity was seen for all time points, suggesting a fixation artefact.

## Discussion

Structural cardiotoxicity is a frequent side effect of TKIs. This study presents, to the best of our knowledge, the largest head-to-head examination of TKI toxicity on contractility in cardiac tissue done so far. Other interesting candidates like trastuzumab were not included due to substantial differences in species specificity [15]. Analysis revealed a decline in contractility for six out of seven cardiotoxic TKIs. This was an encouraging, but imperfect overlap with clinical apportion in cardiotoxic and cardiosafe TKIs. Dasatinib is cardiotoxic in patients but appeared safe with this assay. On the other hand, gefitinib is not considered cardiotoxic but was associated with a decline in EHT contractile function. Potential reasons include species-specific differences in sensitivity. In fact, recent studies on dasatinib cardiotoxicity in rat (H9c2) and human (induced pluripotent stem cell-derived cardiomyocyte) 2D-cell tests revealed a higher sensitivity of the human assay [16,17] and other studies reported a low sensitivity of rat cardiomyocytes for TKI mediated LV dysfunction [7,10]. A recent study in freshly isolated canine cardiomyocytes demonstrated that dasatinib, at a concentration that was without effect in short term incubation and in our assay (10 μM), reduced contractility when incubated for 4 days. Similarly, sunitinib had already effects at 1 μM. The data suggest higher sensitivity of the isolated myocyte assay, which may be due to differences in maturity and species [18], but, conversely, could also indicate higher compensatory capacity of a 3D tissue-like construct. A critical question is how these assays perform in terms of specificity and positive and negative predictive values. This requires the testing of numerous drugs with or without cardiotoxic potential and remains to be done. Immaturity is evident in neonatal cardiac myocytes as present in EHTs, known to exhibit a surprising degree of resistance against hypoxia [19] and immaturity of mitochondria [20], arguing for predominantly anaerobic cellular respiration. This is reducing the impact of mitochondria on cardiomyocyte biology. In fact, studies in H9c2 cell cultures revealed an increase in cardiotoxicity for some TKIs when galactose-containing media was used, resulting in a higher degree of aerobic cellular respiration [16]. However, galactose-based media compositions are not optimized for contractile EHTs so far. Other potential reasons are the relatively short incubation time of this study (4 days), and the use of serum containing cell culture media.

Transmission electron microscopic analysis of subcellular organelles in the presence of TKI revealed a diversity of putative morphological targets (Fig 2B–2F). Among these were alterations in sarcomeres, Z-lines and mitochondria. Furthermore, an increase in autophagolysosomes and residual bodies suggested that autophagy was either activated or autophagic flux was inhibited in the presence of TKIs. A more precise quantification of autophagic activity was performed by LC3 Western immunoblot analysis and determination of LC3-II/I ratio values of the two highest TKI concentrations after 96 hours of incubation (Fig 3). This experiment confirmed the concentration-dependent increase in LC3-II/I ratio values for TKIs.

The severity of contractility decline in this model was expressed as a safety margin, calculated as the ratio between TPC and TTC (Fig 1, S1 Table). Erlotinib and dasatinb were not associated with decline in contractility and led to no or moderate increase in LC3-II/I ratio. TKIs with a smaller SM (gefitinib, lapatinib, sunitinib, imatinib, sorafenib) were also characterized by stronger increase in LC3-II/I ratio values. TKIs with the smallest SM (vandetanib, lestartinib) also had the strongest effect on LC3-II/I ratio (significant increase in LC3-II/I ratio for both tested concentrations). Correlation with SM rank order (S1 Table) revealed that LDH increase was not detected for TKIs with a high SM (erlotinib, dasatinib, gefitinib) and reliably detected for TKIs with a small SM of 10 and below (imatinib, sorafenib, vandetanib, lestartinib), suggesting that LDH release is an indicator for more severe toxicity. Phi coefficient φ correlation revealed a strong association between decline in contractility and LC3-II/I increase (+0.894) and less strong association with LDH- (+0.798) or CK-release (+0.707) (Table 1). Histological analysis of the highest TKI concentrations after 96 hours of incubation supported the contractility data in the sense that the decline in contractility was associated with poor cardiomyocyte morphology (α-sarcomeric actin).

An additional time series experiment was performed with sorafenib, imatinib and sunitinib. The experiments revealed that sarcomeric disarray and damaged mitochondria were already established at the early time points (24–48 hours) for sorafenib and imatinib, but needed 96 hours for sunitinib. This finding was substantiated by caspase-3 staining, demonstrating TKIs to lead to cytoplasmic caspase 3 activity and cellular rarefication (Fig 6). To further dissect the chronology of autophagy impairment, LC3-II/I ratio was analyzed in protein extracts from the same time points. The low LC3-II/I ratio and strong bafilomyocin An effect under (vehicle) control conditions provides evidence for active autophagic flux in control EHTs. In contrast, autophagic flux was maximally inhibited in EHTs in the presence of imatinib, sorafenib and sunitinib, already after 24 hours of incubation (Fig 5B–5G). However, the parallel analysis and similar impairment of autophgic flux of heart cell EHTs and fibroblast EHTs (Fig 5H–5M) demonstrates that TKI mediated impairment of autophagic flux is not specific for cardiomyocytes.

Inhibition of autophagy has been proposed to be mediated by tyrosine kinase inhibitors and evidence was provided in several different experimental models for sorafenib, sunitinib and imatinib [21–26]. Hu et al. suggested that this effect might depend on physicochemical properties based on the observation that analogues of imatinib without kinase inhibitory activity but similar physicochemical properties had cardiotoxic potential. The authors proposed that accumulation of TKIs in lysosomes causes inhibition of autophagic flux and that this causes toxicity [27].

The TKI-mediated early onset autophagy impairment is compatible with autophagy as one mechanism of TKI-cardiotoxicity, but does not prove it. The time course of transmission electron microscopy alterations and the decline in contractility was approximately parallel in the case of sunitinib and imatinib, but not for sorafenib, where force declined after 2 hours. This fast timeline in the latter case argues against autophagy flux inhibition as the primary mechanism of acute toxicity and suggests that effects like kinase inhibition or unidentified effects primarily lead to a decline in contractility in this case (Fig 1A). Thus, the molecular mechanism, and the question as to whether autophagy inhibition is a primary or secondary mechanism of toxicity, requires further studies. Dissecting this mechanism is difficult, since autophagy is a central regulatory pathway with complex interconnections. In particular, autophagy impairment is linked to mitochondrial damage and myofilament disarray, since mitochondrial and sarcomeric integrity strongly depends on functional autophagic flux [28]. Mitochondria are particularly sensitive to autophagy (mitophagy) and impairment is associated with disturbed mitochondrial homeostasis since mitochondria generate, and neutralize, reactive oxygen species. Dysfunction (e.g. during hypoxia) can turn the “power plant” of the cardiomyocytes into producers of excessive reactive oxygen species and up-regulators of pro-death proteins [29].

A limitation of this study is the high TKI concentrations compared to TPC. This discrepancy could be due to several reasons. Tissue concentrations of the highly lipophilic TKI are likely much higher than plasma concentrations. This also means that the toxic threshold in EHTs may have been lower if even longer periods of incubation would have been chosen and drugs would have accumulated to a larger extent. The use of neonatal rat cardiomyocytes and the lack of co-morbidities often present in patients (e.g. increased afterload [7], diabetes and tachycardia) might contribute to the relatively low sensitivity. A second limitation could result from the EHT being less than perfectly supplied by oxygen. This might result in a high turnover of mitochondria and therefore high sensitivity for impairment of auto/mitophagy.

In conclusion, this study demonstrates the feasibility to study TKI-mediated cardiotoxicity in a medium-throughput assay, which is based on contractility and allows secondary analyses such as immunohistochemistry, transmission electron microscopy and Western blot for mechanistic insight. TKIs induced a decline in contractile force in a time and concentration-dependent manner. This was associated with inhibition of autophagic flux. However, this toxicity was not cardiomyocyte-specific. The study paves the way for more detailed and comprehensive studies on molecular mechanisms in human induced pluripotent stem cell-derived EHTs.

## Supporting Information

S1 Fig Experimental outline of the analysis of TKI effects on EHT. (PDF)

S2 Fig TKI effect on EHT contractility. (PDF)

S3 Fig Analysis of lactate dehydrogenase (LDH) and creatin kinase (CK). (PDF)

S4 Fig Electronmicroscopical EHT mosaic images after 24 hours of incubation. (PDF)

S5 Fig Electronmicroscopical EHT mosaic images after 48 hours of incubation. (PDF)

S6 Fig Electronmicroscopical EHT mosaic images after 72 hours of incubation. (PDF)

S7 Fig Electronmicroscopical EHT mosaic images after 96 hours of incubation. (PDF)

S1 Table Summary TKI pharmacology and EHT effects on contractility. (PDF)

S2 Table Morphological alterations of EHT under control. (PDF)

S3 Table Morphological alterations of EHT under vehicle control. (PDF)

S4 Table Morphological alterations of EHT under sorafenib. (PDF)

S5 Table Morphological alterations of EHT under imatinib. (PDF)

S6 Table Morphological alterations of EHT under sunitinib. (PDF)
