# Correction: Analysis of Tyrosine Kinase Inhibitor-Mediated Decline in Contractile Force in Rat Engineered Heart Tissue

**DOI:** 10.1371/journal.pone.0208342

**Published:** 2018-11-27

**Authors:** Fabian Jacob, Amina Y. Yonis, Friederike Cuello, Pradeep Luther, Thomas Schulze, Alexandra Eder, Thomas Streichert, Ingra Mannhardt, Marc N. Hirt, Sebastian Schaaf, Justus Stenzig, Thomas Force, Thomas Eschenhagen, Arne Hansen

The toxic threshold concentration (TTC, μM) values for Sunitinib and Imatinib are swapped in Fig 1B. The toxic threshold concentration for Sunitinib is 1.88 μM and the toxic threshold concentration for Imatinib is 18.99 μM. The safety margin (TTC/TPC) values for Sunitinib and Imatinib are also incorrect. The correct safety margin for Sunitinib is 9.4 and the correct safety margin for Imatinib is 9.4. Please see the correct Fig 1 here.

**Fig 1:**
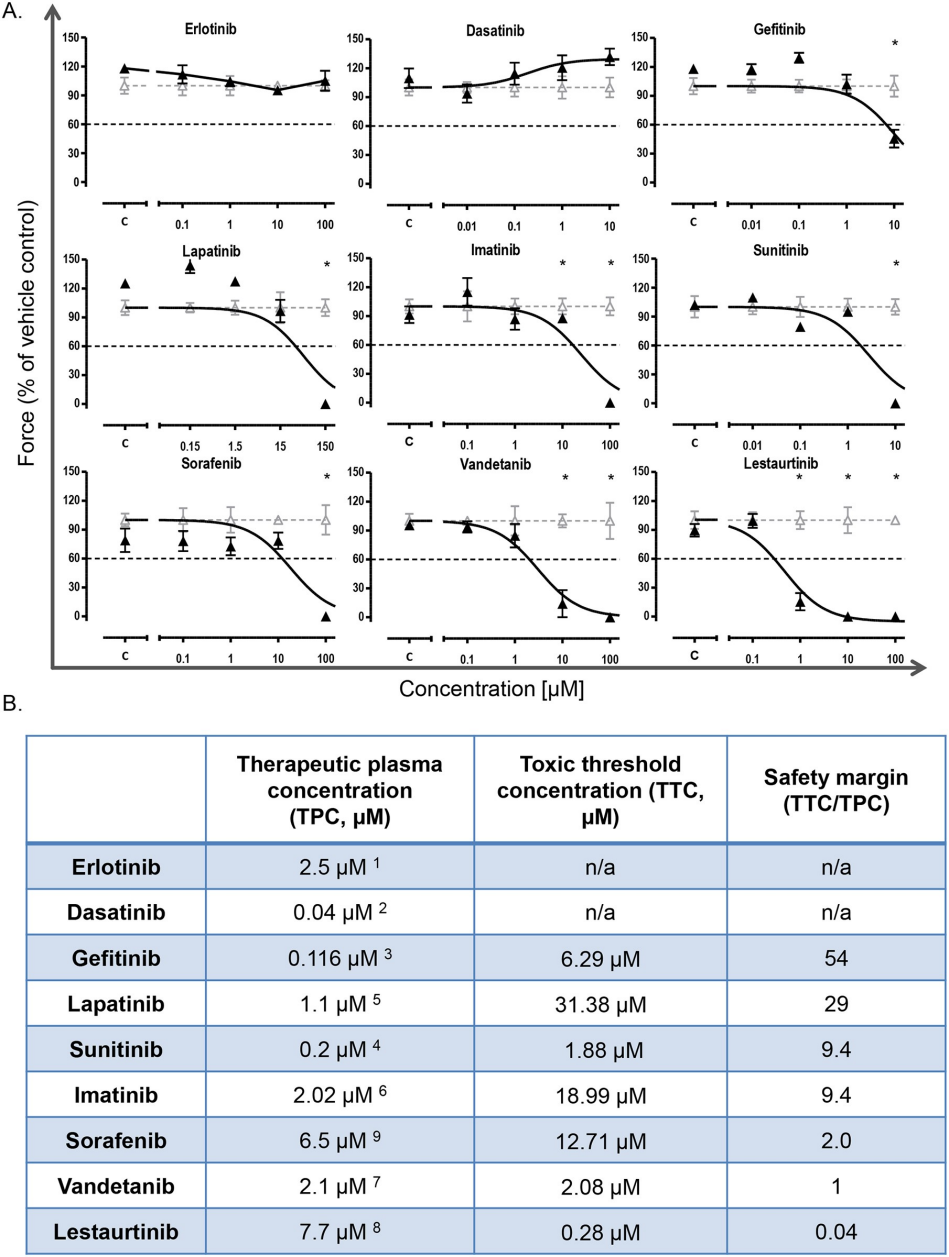
TKI effect on EHT contractility. (A) Depiction of concentration-effect curves (curve-fitted) of 9 TKIs after 96 hours of TKI incubation (▲), normalized to vehicle control (Δ). The toxic threshold (black dashed line) is defined as a decline in contractile force of >40% vs. baseline (BL). Mean values ± SEM; n = 4; *p<0.05 vs. baseline, two-way ANOVA and Bonferroni’s multiple comparison post-test. (B) Total therapeutic plasma concentration (TPC), toxic threshold concentration (TTC: TKI concentration leading to ≥40% reduction in EHT contractile force) and safety margin (SM: TTC/TPC), n/a: not applicable.

The toxic threshold concentration and safety margin values for Sunitinib and Imatinib are also incorrect in S1 Table. Please see the correct S1 Table below.

## Supporting information

S1 Table Summary TKI pharmacology and EHT effects on contractility. (DOCX)
