# Supplementary material for: Analysis of Tyrosine Kinase Inhibitor-Mediated Decline in Contractile Force in Rat Engineered Heart Tissue
Source: PLoS One. 2016 Feb 3;11(2):e0145937. doi: 10.1371/journal.pone.0145937 (PMC4740402; doi:10.1371/journal.pone.0145937)
Supplement: S1 Fig — (PDF) [file pone.0145937.s001.pdf]

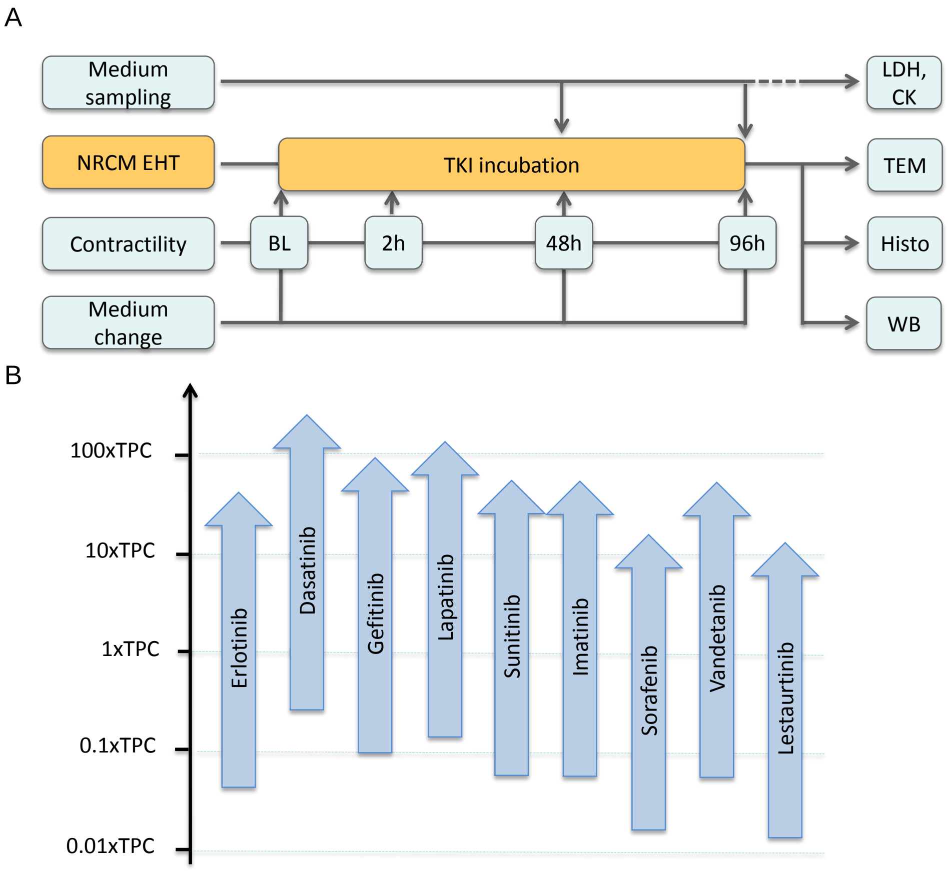

S1 Fig

(A) Experimental outline of the analysis of TKI effects on EHT. BL (baseline), LDH (lactate dehydrogenase), CK (creatin kinase), TEM (transmission electron microscopy), Histo (histology), WB (western blot). (B) Blue arrows indicate the four TKI concentrations (logarithmic steps) analyzed in this study in relation to total therapeutic plasma concentrations (TPC) determined in clinical trials.
