# Supplementary material for: Analysis of Tyrosine Kinase Inhibitor-Mediated Decline in Contractile Force in Rat Engineered Heart Tissue
Source: PLoS One. 2016 Feb 3;11(2):e0145937. doi: 10.1371/journal.pone.0145937 (PMC4740402; doi:10.1371/journal.pone.0145937)
Supplement: S2 Fig — (PDF) [file pone.0145937.s002.pdf]

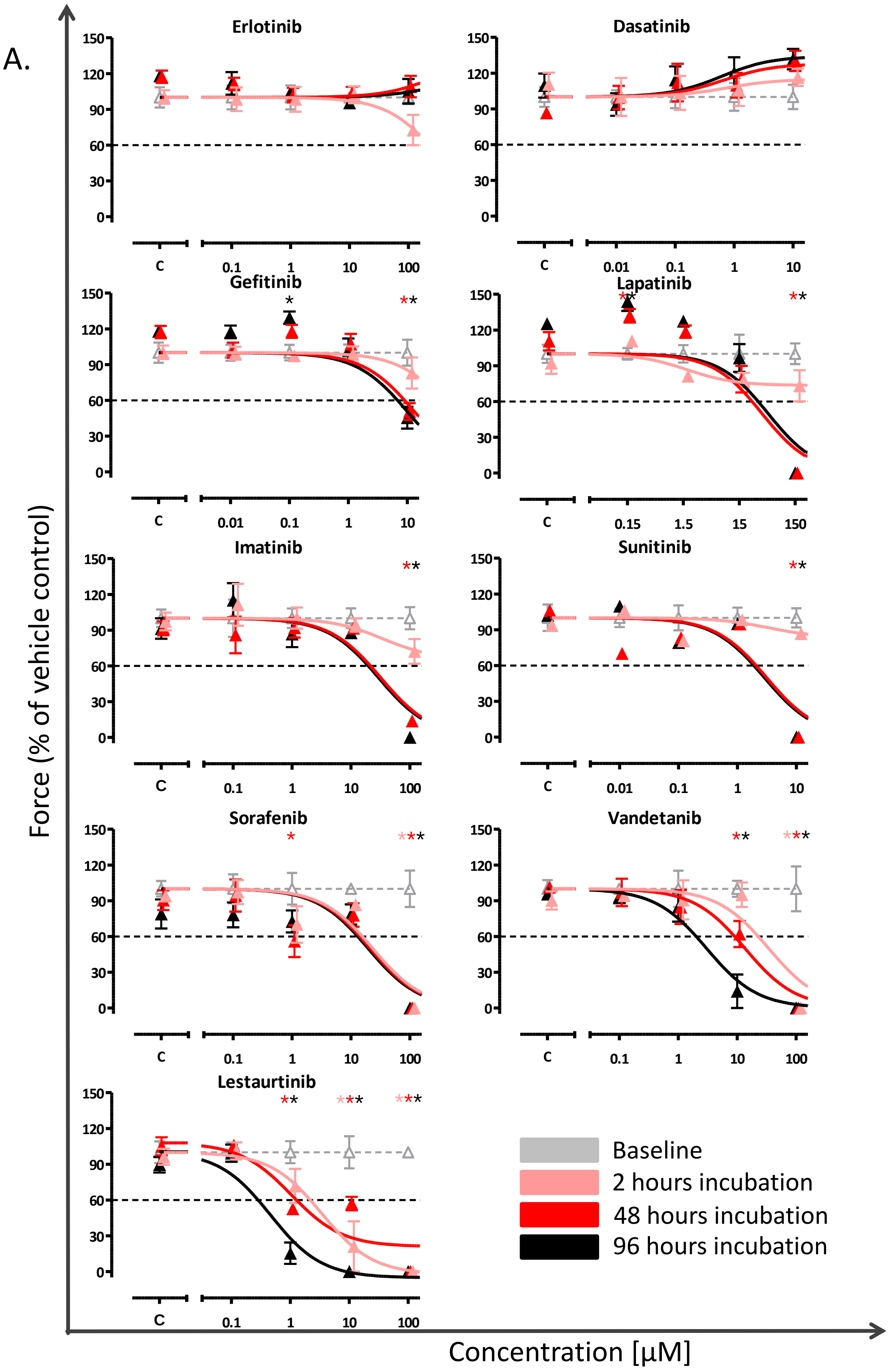
A.
Force (% of vehicle control)
Concentration [µM]
Erlotinib
Dasatinib
Gefitinib
Lapatinib
Imatinib
Sunitinib
Sorafenib
Vandetanib
Lestaurtinib
Baseline
2 hours incubation
48 hours incubation
96 hours incubation

B.

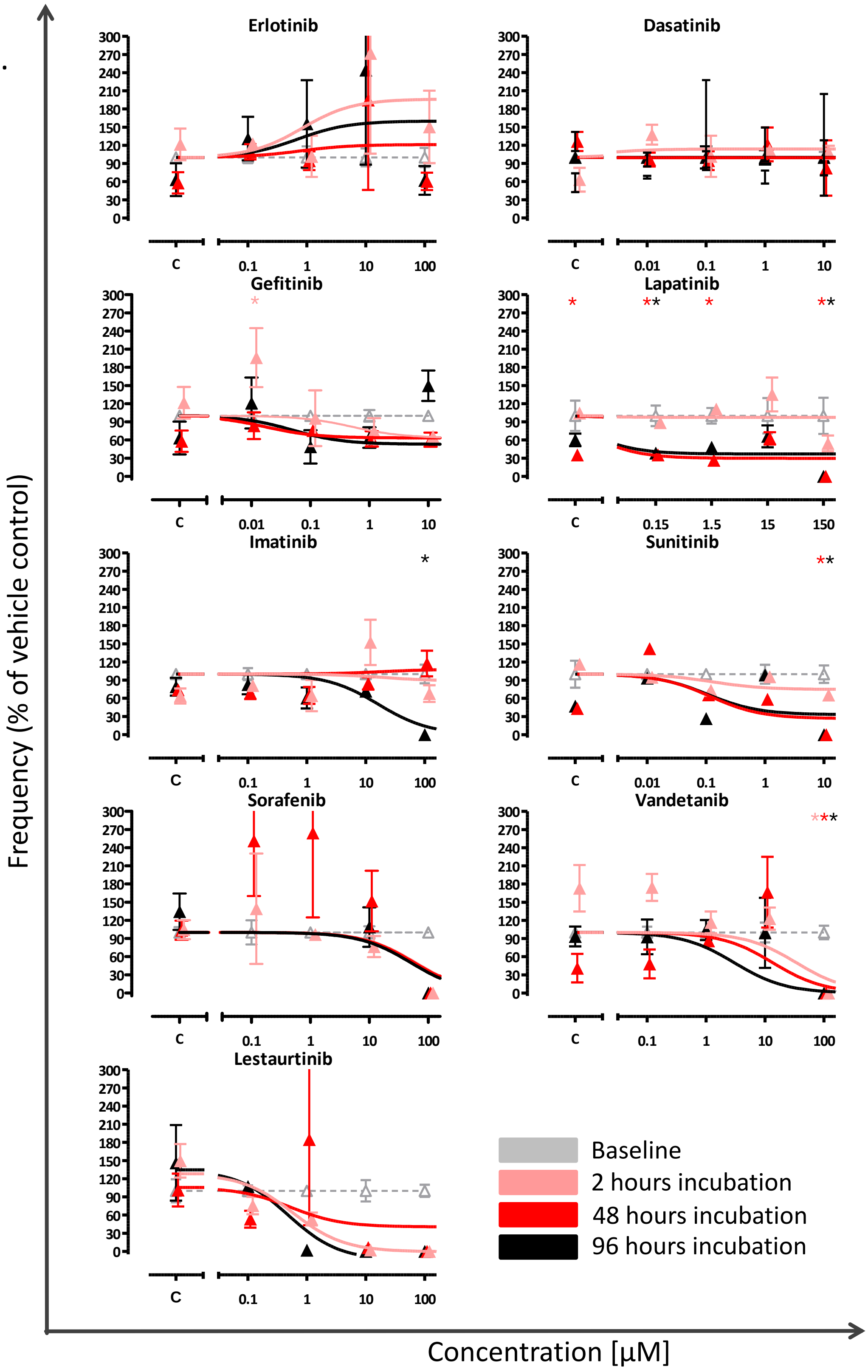
Erlotinib
Dasatinib
Gefitinib
Lapatinib
Imatinib
Sunitinib
Sorafenib
Vandetanib
Lestaurtinib
Baseline
2 hours incubation
48 hours incubation
96 hours incubation
Frequency (% of vehicle control)
Concentration [µM]

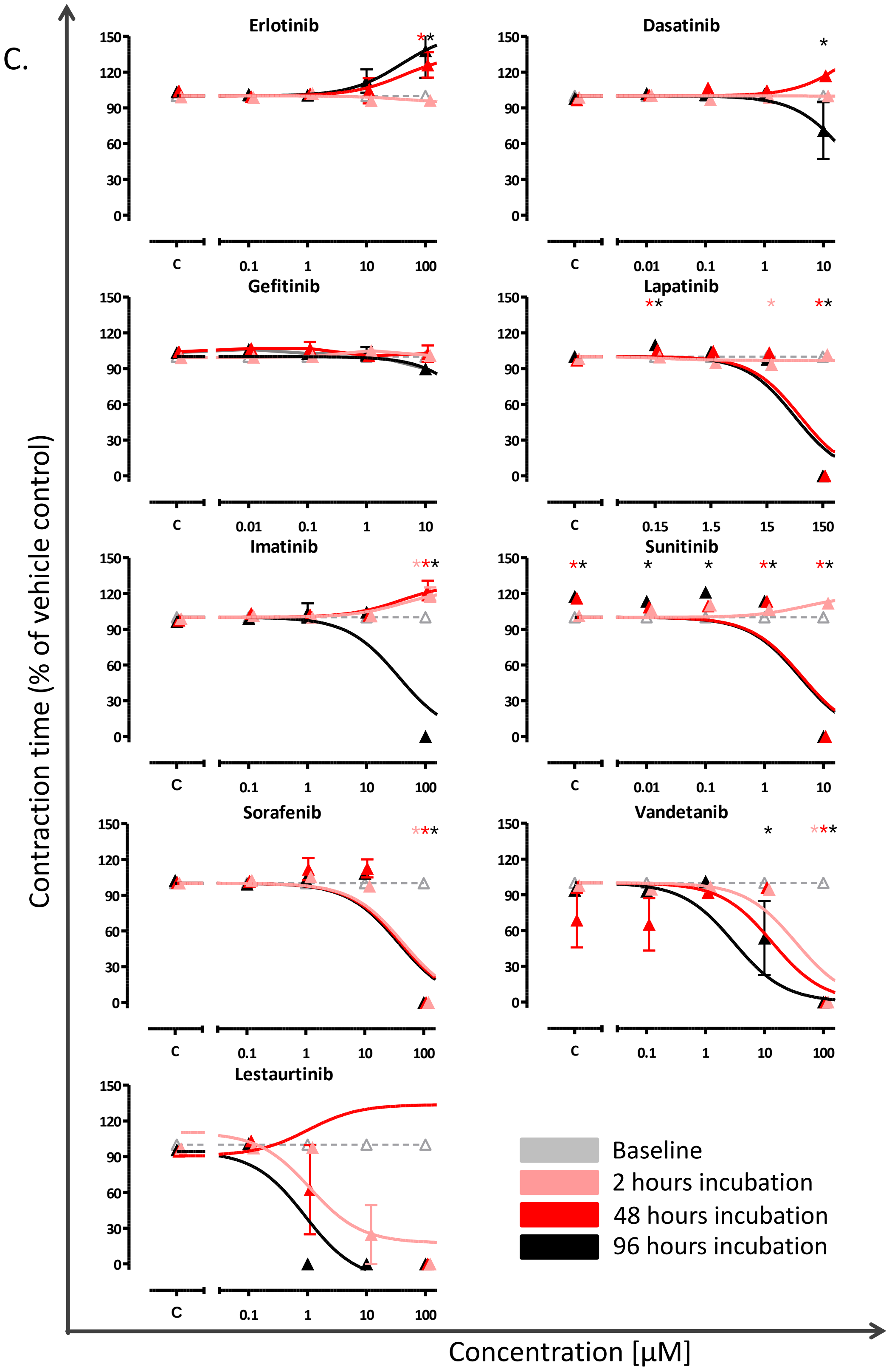
C.
Contraction time (% of vehicle control)
Concentration [µM]
Erlotinib
Dasatinib
Gefitinib
Lapatinib
Imatinib
Sunitinib
Sorafenib
Vandetanib
Lestaurtinib
Baseline
2 hours incubation
48 hours incubation
96 hours incubation

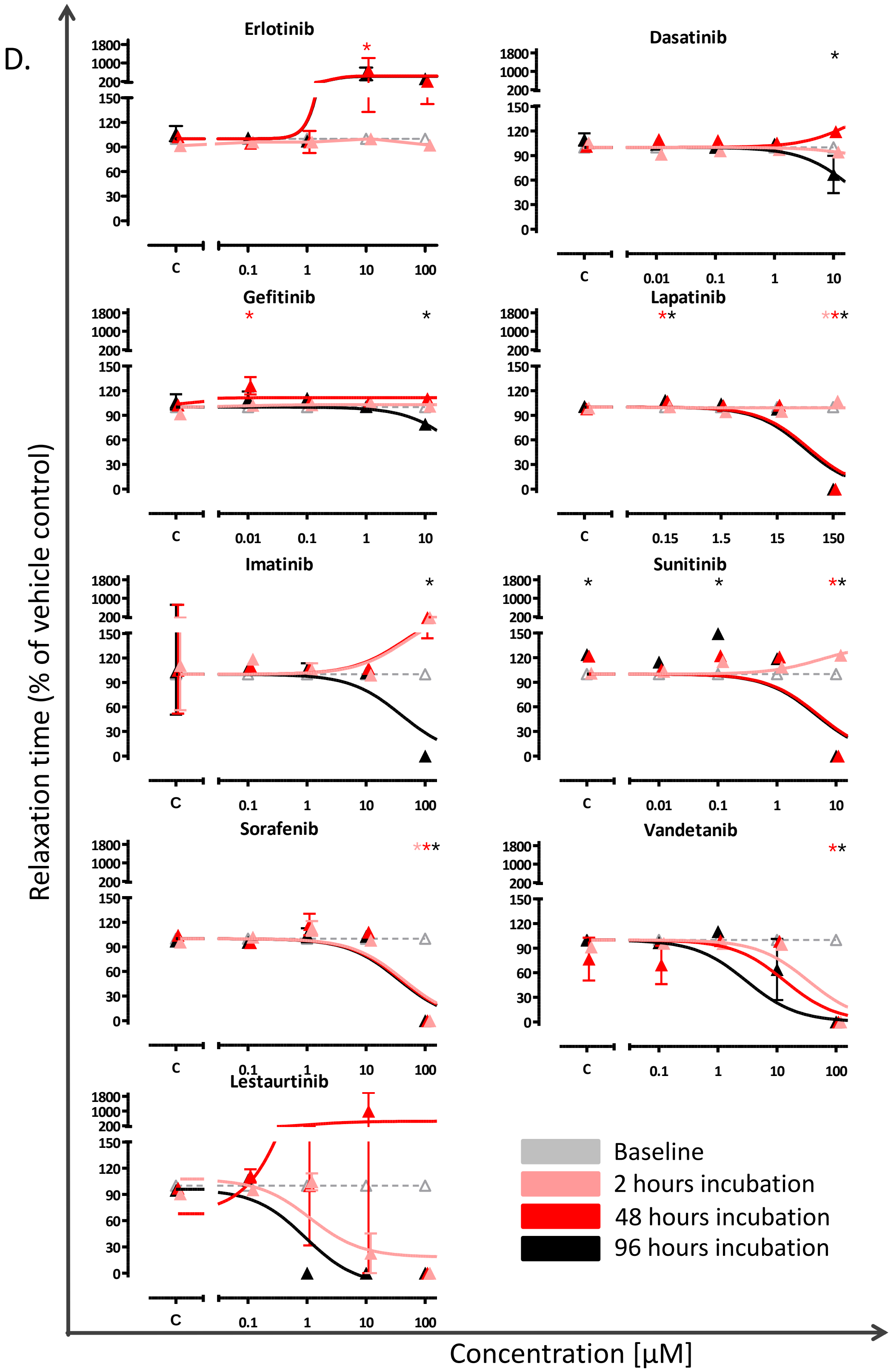

S2 Fig

TKI effect on EHT contractility. Depiction of concentration-effect curves (curve-fitted lines) for force (A), frequency (B), contraction time (C) and relaxation time (D) of 9 TKIs after 2 hours (light red▲), 48 hours (red▲) and 96 hours (black▲) of TKI incubation, normalized to vehicle control (Δ). The toxic threshold (black dashed line) is defined as a decline in contractile force of >40% vs. baseline (BL). Mean values ± SEM; n=4; *p<0.05 vs. vehicle control, two-way ANOVA and Bonferroni's multiple comparison post-test.
