# Supplementary material for: Analysis of Tyrosine Kinase Inhibitor-Mediated Decline in Contractile Force in Rat Engineered Heart Tissue
Source: PLoS One. 2016 Feb 3;11(2):e0145937. doi: 10.1371/journal.pone.0145937 (PMC4740402; doi:10.1371/journal.pone.0145937)
Supplement: S3 Fig — (PDF) [file pone.0145937.s003.pdf]

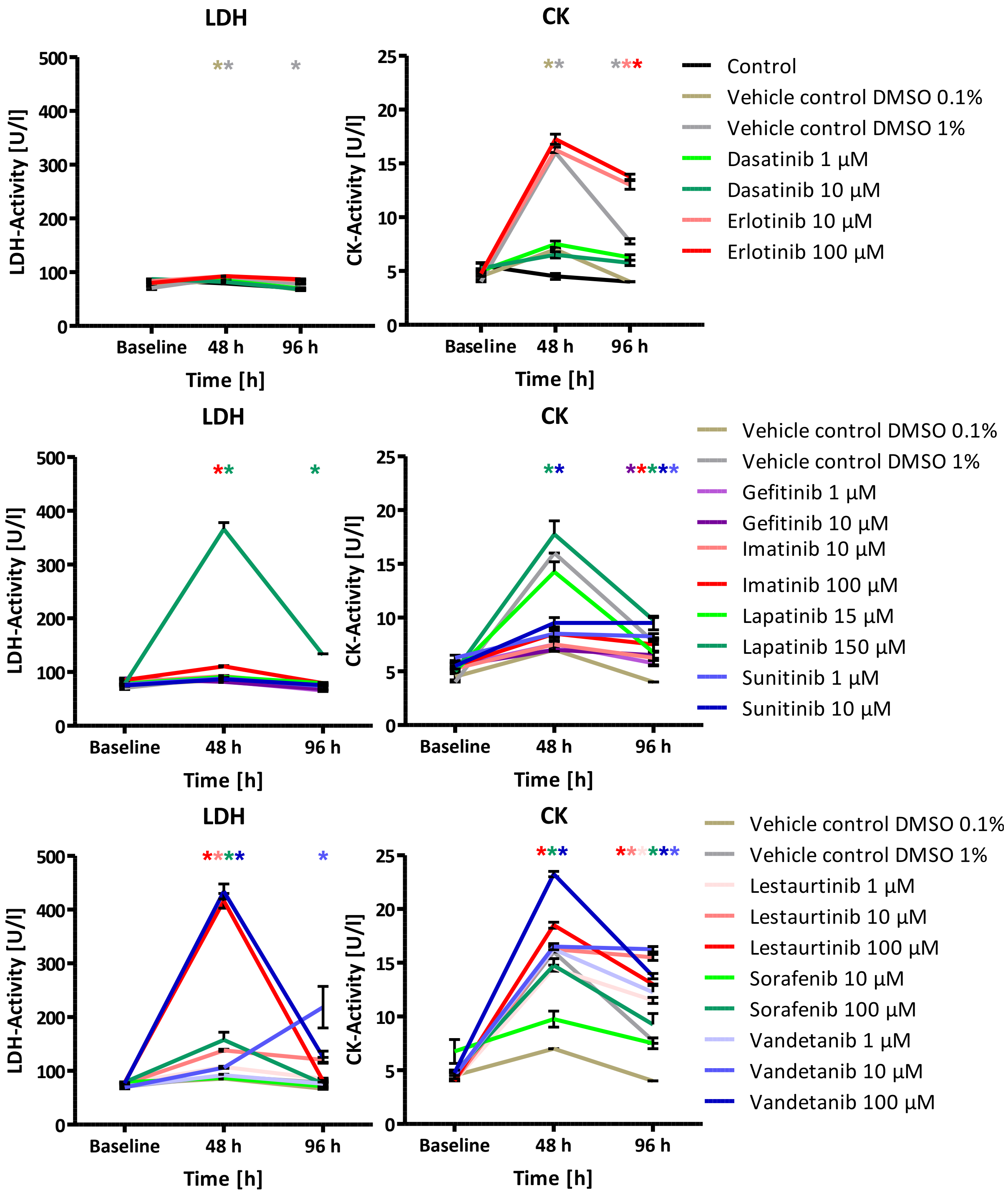

S3 Fig

Analysis of lactate dehydrogenase (LDH) and creatin kinase (CK) in cell culture supernatant of EHT under (vehicle) control condition or in the presence of TKIs after 48 and 96 hours of incubation. Mean values ±SEM; n=4; *$p<0.05$ vs. vehicle control, unpaired student's t-test (two-tailed).
