# Supplementary material for: Analysis of Tyrosine Kinase Inhibitor-Mediated Decline in Contractile Force in Rat Engineered Heart Tissue
Source: PLoS One. 2016 Feb 3;11(2):e0145937. doi: 10.1371/journal.pone.0145937 (PMC4740402; doi:10.1371/journal.pone.0145937)
Supplement: S5 Fig — (PDF) [file pone.0145937.s005.pdf]

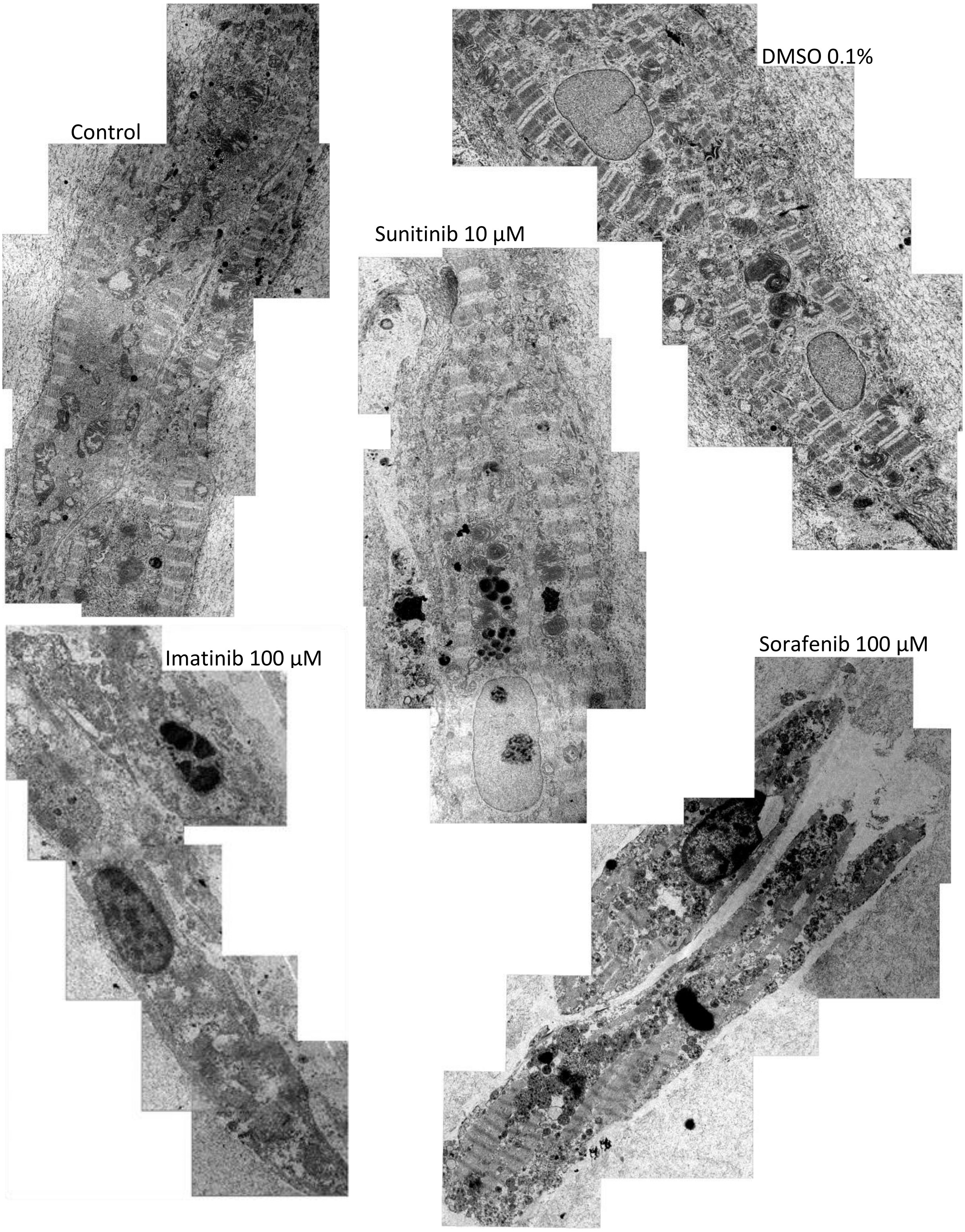

S5 Fig

Electron microscopic EHT mosaic images at 48 hours of incubation under (vehicle) control condition or sunitinib (10 µM), sorafenib (100 µM) or imatinib (100 µM).
