# Supplementary material for: Analysis of Tyrosine Kinase Inhibitor-Mediated Decline in Contractile Force in Rat Engineered Heart Tissue
Source: PLoS One. 2016 Feb 3;11(2):e0145937. doi: 10.1371/journal.pone.0145937 (PMC4740402; doi:10.1371/journal.pone.0145937)
Supplement: S1 Table — (PDF) [file pone.0145937.s008.pdf]

Tyrosin Kinase Inhibitor (TKI)	Trade name	On-targets	Indication (FDA)	Concen-tration range studied on EHTs	Concen-tration range, fold TPC	DSMO Solubility*	DMSO stock concen-tration	Total TPC	TTC	Safety margin (TTC /TPC)
Erlotinib	Tarceva®	ErbB1 (EGFR)	Advanced, metastatic NSCLC, pancreatic cancer	0.1 µM - 100 µM	0.04 – 40x	41 mM	10 mM	2.5 µM[1]	n/a	n/a
Dasatinib	Sprycel®	ABL1/2, c-Kit, PDGFR-α/β SFK	PH+ CML, PH+ ALL	0.01 µM - 10 µM	0.25 - 250x	400 mM	10 mM	0.04 µM[2]	n/a	n/a
Gefitinib	Iressa®	ErbB1 (EGFR)	Advanced, metastatic NSCLC	0.01 µM - 10 µM	0.086 - 86x	224 mM	10 mM	0.116 µM[3]	5.55	54
Lapatinib	Tykerb®	ErbB1 (EGFR), ErbB2 (HER2)	breast cancer ($HER2^+$)	0.15 µM - 150 µM	0.136 - 136x	344 mM	15 mM	1.1 µM[4]	36.1	29
Sunitinib	Sutent®	VEGFR 1-3, c-Kit, PDGFR-α/β, RET, CSF1R, FLT3	RCC, GIST	0.01 µM - 10 µM	0.05 - 50x	75 mM	10 mM	0.2 µM[5]	2.31	95
Imatinib	Gleevec®	ABL 1/2, c-Kit PDGFR-α/β	PH+ CML, PH+ ALL, ASM, Advanced, metastatic DFSP, HES, CEL, advanced, metastatic GIST	0.1 µM - 100 µM	0.05 - 50x	170 mM	100 mM	2.02 µM[6]	20.6	0.9
Sorafenib	Nexavar®	C-Raf, B-Raf, FLT3, VEGFR 1-3, c-Kit PDGFR-α/β	RCC, HCC	0.1 µM - 100 µM	0.015 - 15x	431 mM	100 mM	6.5 µM[7]	16.9	2.0
Vandetanib	Zactima®	ErbB1 (EGFR), VEGFR 2, RET	MTC	0.1 µM - 100 µM	0.05 - 50x	63 mM	10 mM	2.1 µM[8]	2.22	1
Lestaurtinib	-	JAK2, FLT3, RET	Phase II	0.1 µM - 100 µM	0.012 - 12x	227 mM	10 mM	7.7 µM[9]	0.297	0.04

S1 Table

Summary TKI pharmacology and EHT effects on contractility. Therapeutic plasma concentrations (TPC), toxic threshold concentration (TTC), ABL1/2 (abelson murine leukemia viral oncogene homolog 1/2), AMPK (5' AMP-activated protein kinase), B-Raf (B-rapidly growing fibrosarcoma), c-Kit (stem cell factor receptor), C-Raf (C-rapidly growing fibrosarcoma), CEL (chronic

eosinophilic leukaemia) CML (chronic myeloid leukaemia), CSF1R (colony-stimulating factor 1 receptor), EGFR (epidermal growth factor receptor), ErbB2 (human epidermal growth factor receptor 2, HER2), ErbB4 (human epidermal growth factor receptor 4), FLT3 (FMS-related tyrosine kinase 3), GIST (gastrointestinal stromal tumour), HCC (hepatocellular carcinoma), HES (hypereosinophilic syndrome), JAK2 (Januskinase 2), MTC (medullary thyroid cancer), mTOR (mammalian target of rapamycin), NSCLC (non-small-cell lung cancer), PDGFR (platelet-derived growth factor receptor), PH+ (Philadelphia chromosome positive), RCC (renal cell carcinoma), VEGFR (vascular endothelial growth factor receptor); Aggressiv systemic mastocytosis (ASM), dematofibrosarcoma protuberans (DFSP), gastrotintestinal stromal tumor (GIST) (Adopted from[10])
n/a: non applicable

*DMSO solubility is listed as specified in the vendors' data sheet.

References:

1. Soulieres D, Senzer NN, Vokes EE, Hidalgo M, Agarwala SS, Siu LL. Multicenter phase II study of erlotinib, an oral epidermal growth factor receptor tyrosine kinase inhibitor, in patients with recurrent or metastatic squamous cell cancer of the head and neck. *J Clin Oncol*. 2004;22:77–85.

2. Demetri GD, Lo Russo P, MacPherson IRJ, Wang D, Morgan J a, Brunton VG, Paliwal P, Agrawal S, Voi M, Evans TRJ. Phase I dose-escalation and pharmacokinetic study of dasatinib in patients with advanced solid tumors. *Clin Cancer Res*. 2009;15:6232–40.

3. Baselga J. Phase I Safety, Pharmacokinetic, and Pharmacodynamic Trial of ZD1839, a Selective Oral Epidermal Growth Factor Receptor Tyrosine Kinase Inhibitor, in Patients With Five Selected Solid Tumor Types. *J Clin Oncol*. 2002;20:4292–4302.

4. Burris H a, Hurwitz HI, Dees EC, Dowlati A, Blackwell KL, O'Neil B, Marcom PK, Ellis MJ, Overmoyer B, Jones SF, Harris JL, Smith D a, Koch KM, Stead A, Mangum S, Spector NL. Phase I safety, pharmacokinetics, and clinical activity study of lapatinib (GW572016), a reversible dual inhibitor of epidermal growth factor receptor tyrosine kinases, in heavily pretreated patients with metastatic carcinomas. *J Clin Oncol*. 2005;23:5305–13.

5. Faivre S, Delbaldo C, Vera K, Robert C, Lozahic S, Lassau N, Bello C, Deprimo S, Brega N, Massimini G, Armand J-P, Scigalla P, Raymond E. Safety, pharmacokinetic, and antitumor activity of SU11248, a novel oral multitarget tyrosine kinase inhibitor, in patients with cancer. *J Clin Oncol*. 2006;24:25–35.

6. Cortes JE, Egorin MJ, Guilhot F, Molimard M, Mahon F-X. Pharmacokinetic/pharmacodynamic correlation and blood-level testing in imatinib therapy for chronic myeloid leukemia. *Leukemia*. 2009;23:1537–44.

7. Clark JW, Eder JP, Ryan D, Lathia C, Lenz H. Vascular Endothelial Growth Factor Receptor Inhibitor , BAY 43-9006 , in Patients with Advanced , Refractory Solid Tumors. 2005;11:5472–5480.

8. Holden SN, Eckhardt SG, Basser R, de Boer R, Rischin D, Green M, Rosenthal M a, Wheeler C, Barge a, Hurwitz HI. Clinical evaluation of ZD6474, an orally active inhibitor of VEGF and EGF receptor signaling, in patients with solid, malignant tumors. *Ann Oncol*. 2005;16:1391–7.

9. Levis M, Ravandi F, Wang ES, Baer MR, Perl A, Coutre S, Erba H, Stuart RK, Baccarani M, Cripe LD, Tallman MS, Meloni G, Godley L a, Langston A a, Amadori S, Lewis ID, Nagler A, Stone R, Yee K, Advani A, Douer D, Wiktor-Jedrzejczak W, Juliusson G, Litzow MR, Petersdorf S, Sanz M, Kantarjian HM, Sato T, Tremmel L, Bensen-Kennedy DM, Small D, Smith BD. Results from a randomized trial of salvage chemotherapy followed by lestaurtinib for patients with FLT3 mutant AML in first relapse. *Blood*. 2011;117:3294–301.

10. Force T, Kolaja KL. Cardiotoxicity of kinase inhibitors: the prediction and translation of preclinical models to clinical outcomes. *Nat Rev Drug Discov*. 2011;10:111–26.
