# Supplementary material for: Analysis of Tyrosine Kinase Inhibitor-Mediated Decline in Contractile Force in Rat Engineered Heart Tissue
Source: PLoS One. 2016 Feb 3;11(2):e0145937. doi: 10.1371/journal.pone.0145937 (PMC4740402; doi:10.1371/journal.pone.0145937)
Supplement: S2 Table — (PDF) [file pone.0145937.s009.pdf]

	Light Microscopy	Mitochondria	Z-line	Disarray	Junctions	Autophagy	Myelin bodies	Contracted/ relaxed
24 hours	Regularity in sarcomeric structure, well-defined striations – no disruptions	Majority normal sized/ shaped (some irregularity)	Well-defined + sharp	None	Ok – gap junctions, desmosomes + fascia adherens visible	None	None	Mixed (large contracted regions)
48 hours	Well-defined regular sarcomere organisation – no disruption	Some variation in shape – some very small sized	Well-defined + sharp	Minimal	Desmosomes visible	None	Large circular bodies - grouped	Relaxed
72 hours	Shorter myofibrils generally, regular sarcomere structures visible	Groups of very small mitochondria + individual large mitochondria with damaged cristae	Well-defined + sharp	None	Ok – gap junctions, desmosomes visible	None	Large circular	Relaxed
96 hours	Majority regular structures with centralised nucleus, some autophagy bodies seen. Mostly well defined striations	Mixture of sizes and shapes, some very large whilst others small – some exhibit damaged cristae	Generally defined	Minimal	Ok – gap junctions & desmosomes regular	Some autophagy evident through residual bodies	Large circular	Mixture
24 hours	Well-defined sarcomeres with centralised nucleus. Some irregularities visible	Groups of mitochondria with damaged cristae	Generally defined (some regions of disarray)	Some sarcomere disarray	Ok – desmosomes visible	Some residual bodies visible	None	Relaxed

S2 Table. Morphological alterations of EHT under control conditions (24-96 hours), 1st column (red heading): light microscopy observations; 2-9th columns (blue headings): Electron microscopy observations.
