# Supplementary material for: Analysis of Tyrosine Kinase Inhibitor-Mediated Decline in Contractile Force in Rat Engineered Heart Tissue
Source: PLoS One. 2016 Feb 3;11(2):e0145937. doi: 10.1371/journal.pone.0145937 (PMC4740402; doi:10.1371/journal.pone.0145937)
Supplement: S3 Table — (PDF) [file pone.0145937.s010.pdf]

	Light Microscopy	Mitochondria	Z-line	Disarray	Junctions	Autophagy	Myelin bodies	Contracted/ relaxed
24 hours	Generally well-defined regular sarcomere organisation, some irregularities	Damaged cristae – various shapes/sizes	Well-defined	Some regions of disarray	Ok – gap junction + desmosome visible	None	Circular bodies	Relaxed
48 hours	More irregularities, large areas of defined sarcomere organisation visible	Mixture of large and very small mitochondria – damaged cristae in large mitochondria	Well-defined + sharp	None	Ok – gap junctions, desmosomes + fascia adherens visible	None	Large circular body	Relaxed
72 hours	Minimal disarray, mostly clear regular striations visible – long myofibrils, with centralised nucleus	Large mitochondria, some with damaged cristae	Well-defined generally	Minimal	Ok	Very minimal	Yes, circular body	Relaxed
96 hours	Continuous regions of defined structure visible, some disruption	Varying shapes and sizes with cristae damage	Well-defined + sharp	None	Ok – gap junction + desmosome visible	Very minimal	Circular bodies	Relaxed

S3 Table. Morphological alterations of EHT under vehicle control conditions (DMSO 0.1; 24-96 hours), 1st column (red heading): light microscopy observations; 2-9th columns (blue headings): Electron microscopy observations.
