# Supplementary material for: Analysis of Tyrosine Kinase Inhibitor-Mediated Decline in Contractile Force in Rat Engineered Heart Tissue
Source: PLoS One. 2016 Feb 3;11(2):e0145937. doi: 10.1371/journal.pone.0145937 (PMC4740402; doi:10.1371/journal.pone.0145937)
Supplement: S4 Table — (PDF) [file pone.0145937.s011.pdf]

	Light Microscopy	Mitochondria	Z-line	Disarray	Junctions	Autophagy	Myelin bodies	Contracted/ relaxed
24 hours	Sparse organisation with a lack of striations and large disruption	Small with damaged cristae, some intact mitochondria	Some ragged	Some disarray	Ok – gap junction + desmosomes visible	Yes – numerous residual bodies visible	None	Mostly contracted (little relaxed regions)
48 hours	Circular bodies with no sarcomeric structure, large disruption	No intact mitochondria	None visible	Complete disarray	Irregular desmosomes – very short and random	Large areas of residual bodies	None	Contracted – hard to tell
72 hours	Irregular shaped nuclei, lack of striations or regularity	Small irregular mitochondria, lack of integrity/ structure, extremely damaged cristae	Not visible	Complete disarray, lack of structural integrity	Very long gap junction, fat desmosomes	Widespread residual bodies	None	Contracted
96 hours	Nuclei near outer edge – no structural integrity, lack of sarcomere/ striations	Groups of very small damaged mitochondria – hard to distinguish	Very thick Z-line and misaligned	Complete disarray	Intact very long gap junction, other short desmosomes visible	Large density of residual bodies	None	Contracted

S4 Table. Morphological alterations of EHT under sorafenib (100 µM; 24-96 hours), 1st column (red heading): light microscopy observations; 2-9th columns (blue headings): Electron microscopy observations.
