# Supplementary material for: Analysis of Tyrosine Kinase Inhibitor-Mediated Decline in Contractile Force in Rat Engineered Heart Tissue
Source: PLoS One. 2016 Feb 3;11(2):e0145937. doi: 10.1371/journal.pone.0145937 (PMC4740402; doi:10.1371/journal.pone.0145937)
Supplement: S5 Table — (PDF) [file pone.0145937.s012.pdf]

	Light Microscopy	Mitochondria	Z-line	Disarray	Junctions	Autophagy	Myelin bodies	Contracted/ relaxed
24 hours	Small regions of sarcomere/ striation organisation, numerous regions of disarray and irregularity	Varying shapes of damaged mitochondria spread between large cytoplasmic space	Ragged at places and completely damaged in others	Mixture between array and disarray – more disarray	Some desmosomes – randomly placed and irregular	Yes – small number of residual bodies seen	Circular regular body	Relaxed (very little contracted region)
48 hours	Very little area of sarcomere regularity, loss of organisation – irregular shaped nuclei	Various shapes with irregularity – lack of structure, damaged cristae	Ragged	Complete disarray	Irregular desmosomes – very small	Complete autophagy with many residual bodies	None	Mixed
72 hours	Very small circular nucleus, no striations visible and loss of organisation	Completely defaced and damaged, lack of integrity/ structure	Disarrayed and hard to recognise	Complete disarray	Small desmosomes distinguishable and thin gap junctions	Mostly autophagy, numerous residual bodies	None	Only contracted regions are recognisable
96 hours	Complete oblivion of structured sarcomeres, complete lack of striations	All exhibiting damaged cristae with lack of structural integrity, small and random dispersion	Thick and irregular – merged into large dark ball	Complete disarray – no structural definition	Very small desmosomes – damaged end structure, gap junction seen intact	Completely residual bodies	None	Contracted – very little visible and hard to distinguish

S5 Table. Morphological alterations of EHT under imatinib (100 µM; 24-96 hours), 1st column (red heading): light microscopy observations; 2-9th columns (blue headings): Electron microscopy observations.
