# Supplementary material for: Analysis of Tyrosine Kinase Inhibitor-Mediated Decline in Contractile Force in Rat Engineered Heart Tissue
Source: PLoS One. 2016 Feb 3;11(2):e0145937. doi: 10.1371/journal.pone.0145937 (PMC4740402; doi:10.1371/journal.pone.0145937)
Supplement: S6 Table — (PDF) [file pone.0145937.s013.pdf]

	Light Microscopy	Mitochondria	Z-line	Disarray	Junctions	Autophagy	Myelin bodies	Contracted/ relaxed
24 hours	Plenty of regular sarcomere regions with centralised nucleus	Round, regular shapes – damaged cristae, some very long	Aligned mostly	Some disarray	Desmosomes visible – very 'fat' + irregular central region	Yes – large residual bodies visible	None	Relaxed
48 hours	Introduction of irregularity and disruption becomes more pronounced	Some intact mitochondria, extremely large	Some ragged regions	Some disarray	Very small desmosomes – thin border, intact gap junction	Yes – large + small residual bodies visible	Irregular body	Relaxed
72 hours	Regular striations visible, some regions of disarray	Large, some intact – some with damaged cristae	Areas of ragged z-lines, some aligned	Some disarray evident	Long thin gap junctions, small desmosomes visible	Some autophagy	None	Relaxed
96 hours	Complete disruption (possibly the worst of the three drugs), no structure at all, hard to distinguish structural regions	None intact	Indistinguishable	Large disarray	Irregular desmosomes, some gap junctions	Numerous residual bodies visible	None	Contracted

S6 Table. Morphological alterations of EHT under sunitinib (10 µM; 24-96 hours), 1st column (red heading): light microscopy observations; 2-9th columns (blue headings): Electron microscopy observations.
